# Survival and abundance of polar bears in Alaska’s Beaufort Sea, 2001–2016

**DOI:** 10.1002/ece3.8139

**Published:** 2021-09-23

**Authors:** Jeffrey F. Bromaghin, David C. Douglas, George M. Durner, Kristin S. Simac, Todd C. Atwood

**Affiliations:** ^1^ U.S. Geological Survey Alaska Science Center Anchorage AK USA; ^2^ U.S. Geological Survey Alaska Science Center Juneau AK USA

**Keywords:** Arctic, Bayesian hierarchical model, climate warming, mark–recapture, multistate, Ursus maritimus

## Abstract

The Arctic Ocean is undergoing rapid transformation toward a seasonally ice‐free ecosystem. As ice‐adapted apex predators, polar bears (*Ursus maritimus*) are challenged to cope with ongoing habitat degradation and changes in their prey base driven by food‐web response to climate warming. Knowledge of polar bear response to environmental change is necessary to understand ecosystem dynamics and inform conservation decisions. In the southern Beaufort Sea (SBS) of Alaska and western Canada, sea ice extent has declined since satellite observations began in 1979 and available evidence suggests that the carrying capacity of the SBS for polar bears has trended lower for nearly two decades. In this study, we investigated the population dynamics of polar bears in Alaska's SBS from 2001 to 2016 using a multistate Cormack–Jolly–Seber mark–recapture model. States were defined as geographic regions, and we used location data from mark–recapture observations and satellite‐telemetered bears to model transitions between states and thereby explain heterogeneity in recapture probabilities. Our results corroborate prior findings that the SBS subpopulation experienced low survival from 2003 to 2006. Survival improved modestly from 2006 to 2008 and afterward rebounded to comparatively high levels for the remainder of the study, except in 2012. Abundance moved in concert with survival throughout the study period, declining substantially from 2003 and 2006 and afterward fluctuating with lower variation around an average of 565 bears (95% Bayesian credible interval [340, 920]) through 2015. Even though abundance was comparatively stable and without sustained trend from 2006 to 2015, polar bears in the Alaska SBS were less abundant over that period than at any time since passage of the U.S. Marine Mammal Protection Act. The potential for recovery is likely limited by the degree of habitat degradation the subpopulation has experienced, and future reductions in carrying capacity are expected given current projections for continued climate warming.

## Graphical abstract

As ice‐adapted apex predators, polar bears (*Ursus maritimus*) are challenged to cope with the ongoing habitat degradation and changes in their prey base driven by food web response to climate warming. We investigated the population dynamics of polar bears in Alaska's southern Beaufort Sea (SBS) using a multistate CJS mark–recapture model. Abundance declined during several consecutive years of poor survival and afterward fluctuated in concert with survival but without sustained trend, averaging 565 bears from 2006 to 2015, the lowest level since passage of the US Marine Mammal Protection Act.

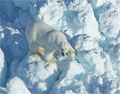

## 1 INTRODUCTION

The Arctic continues to undergo profound change at a rapid pace. Average annual air temperature has increased by nearly 3°C in the last five decades, a rate of change more than double that of the Northern Hemisphere overall (Box et al., 2019), and episodes of unseasonably warm midwinter temperatures are becoming more frequent (Graham et al., 2017; Moore, 2016a). Climate warming has led to rapid reductions in sea ice extent, volume, and age (e.g., Kwok, 2018), and peripheral Arctic seas are either seasonally ice‐free or projected to become so within a decade (Onarheim et al., 2018). The loss of stabilizing sea ice cover has intensified geophysical processes that are expected to induce greater ice loss in the future (Post et al., 2019), including increased intrusion of warm sub‐Arctic waters (Barton et al., 2018; Pacini et al., 2019; Wang et al., 2020) and vertical mixing (Liang & Losch, 2018), greater absorption of solar radiation by ice‐free waters (Timmermans et al., 2018), and increased wind speed and wave height (Li et al., 2019; Liu et al., 2016) with corresponding ice deformation (Lei et al., 2020; Williams et al., 2013; Zhang et al., 2012).

The transformation of abiotic processes in the Arctic Ocean creates both direct and indirect forcing at all levels of the biota. Primary productivity is increasing (Jin et al., 2016) through an earlier spring bloom and a longer period of production (Kahru et al., 2016), including more frequent fall blooms (Waga & Hirawake, 2020) fueled by nutrients made available by wind‐induced shelf upwelling (Pickart et al., 2013; Schulze & Pickart, 2012). Changes in the source and timing of primary production may cascade to higher trophic levels and alter the species composition of zooplankton and tertiary consumers (Ehrlich et al., 2020; Fujiwara et al., 2018; Hop et al., 2020; Spear et al., 2019), with a resulting shift from benthic to pelagic production (Grebmeier et al., 2006). Earlier ice melt has also been associated with enhanced ringed seal (*Pusa hispida*) growth and productivity in the eastern Beaufort Sea (Harwood et al., 2020; Nguyen et al., 2017). The community of fish species in the Atlantic sector of the Arctic is transitioning from small‐bodied, demersal species to larger, generalist species more typical of boreal waters (Frainer et al., 2017). The seabird community in the Chukchi Sea is now dominated by planktivorous species, seemingly in response to increased zooplankton production (Gall et al., 2017). Sub‐Arctic baleen whales and killer whales (*Orcinus orca*) are also becoming more abundant in the Arctic (Higdon & Ferguson, 2009; Moore, 2016b), and bowhead whales (*Balaena mysticetus*) were recently documented to overwinter in the eastern Beaufort Sea (Insley et al., 2021). Overall, Arctic ecosystems may be reorganizing in response to rapid environmental change (Grebmeier, 2012; Huntington et al., 2020).

As an ice‐dependent apex predator, polar bears (*Ursus maritimus*) are directly affected by the availability and physical structure of sea ice and changes in the accessibility or quality of their prey as the Arctic ecosystem responds to the warming climate. However, the Arctic is not homogeneous throughout and each polar bear subpopulation is subject to the forcing factors operating within its range. The Chukchi Sea subpopulation, for example, appears to remain healthy despite experiencing considerable summer sea ice loss, presumably because ecosystem productivity is high and access to prey remains adequate (Regehr et al., 2018; Rode et al., 2014, 2018, 2021). Indeed, some have speculated that a reduction in the prevalence of thick, multiyear ice may increase ecosystem productivity and thereby provide short‐term benefit to high‐Arctic subpopulations (Derocher et al., 2004), although evidence regarding that hypothesis is limited (Florko et al., 2021; Laidre et al., 2020b). Even so, the long‐term effects of climate warming for the species can only be negative (Atwood, Marcot, et al., 2016; Molnár et al., 2020; Stirling & Derocher, 2012). Several investigators have documented population‐level response to ecosystem change, many with potentially negative consequences, including shifts in the distribution of maternal dens and shorter den occupancy (Escajeda et al., 2018; Olson et al., 2017), greater use of terrestrial habitats during summer (Atwood, Peacock, et al., 2016; Laidre et al., 2020a; Rode et al., 2015; Ware et al., 2017), more frequent swimming bouts and elevated energy expenditure (Durner et al., 2017; Lone et al., 2018; Pagano et al., 2012), higher rates of fasting in the spring (Rode et al., 2018), and reductions in body condition, reproductive output, and survival (Laidre et al., 2020a; Obbard et al., 2016; Rode et al., 2010). Increased use of terrestrial habitats has been associated with elevated immune response and altered microbiota with unknown long‐term consequences (Atwood et al., 2017; Watson et al., 2019; Whiteman et al., 2019). Although ringed and bearded (*Erignathus barbatus*) seals remain the primary prey for polar bears throughout most of the Arctic (Derocher et al., 2002; Florko et al., 2020; Galicia et al., 2015; McKinney et al., 2017; Thiemann et al., 2008), ringed seal body condition is declining in at least one region of the Canadian Archipelago (Harwood et al., 2020) and ice loss and shifts in species composition have led to greater use of alternative prey species by some subpopulations (Florko et al., 2021; Galicia et al., 2015, 2016; Iverson et al., 2014; Laidre et al., 2018).

The Southern Beaufort Sea (SBS) subpopulation of polar bears is one of the few subpopulations with long‐term research sufficient to document population response to climate warming and sea ice loss. The subpopulation is thought to have been overharvested prior to the passage of the Marine Mammal Protection Act (MMPA) in 1972, based primarily on a combination of temporal trends in bear encounter rates during small aircraft flights and a steady decline in the average age of harvested bears (Amstrup et al., 1986). Early mark–recapture research results estimated subpopulation abundance to average 1,776 bears in the decade after passage of the MMPA (Amstrup et al., 1986). A later analysis of female data collected through 1998 concluded that subpopulation size trended higher through the 1980s and into the late 1990s, likely exceeding 2,000 bears though total abundance levels were somewhat uncertain (Amstrup et al., 2001). A renewed focus on mark–recapture research was initiated in 2001, and an analysis of data collected from 2001 to 2010 found that subpopulation abundance was 1,607 bears (90% confidence interval [836, 2379]) in 2004 and low survival from 2004 through at least 2007 led to additional reductions, with an estimated abundance of 907 [303, 1,511] bears in 2010 (Bromaghin et al., 2015).

In this study, we analyzed 2001–2016 mark–recapture and telemetry data collected from the SBS polar bear subpopulation along Alaska's north coast, an area that encompasses a majority of the SBS subpopulation range. Our primary objective was to assess subpopulation status through estimation of survival rates and abundance, particularly in comparison with the relative stability from 2007 to 2010 reported by Bromaghin et al. (2015). A secondary objective was to evaluate the potential for spatial information to account for heterogeneity in polar bear recapture probabilities, compared with prior models using covariates for this purpose, and thereby improve model structure and the resulting estimates of vital rates and abundance. We summarize the resulting spatial model and present an updated assessment of the recent population dynamics of the SBS polar bear subpopulation in Alaska.

## 2 MATERIALS AND METHODS

### 2.1 Data collection and preparation

**FIGURE 1:**
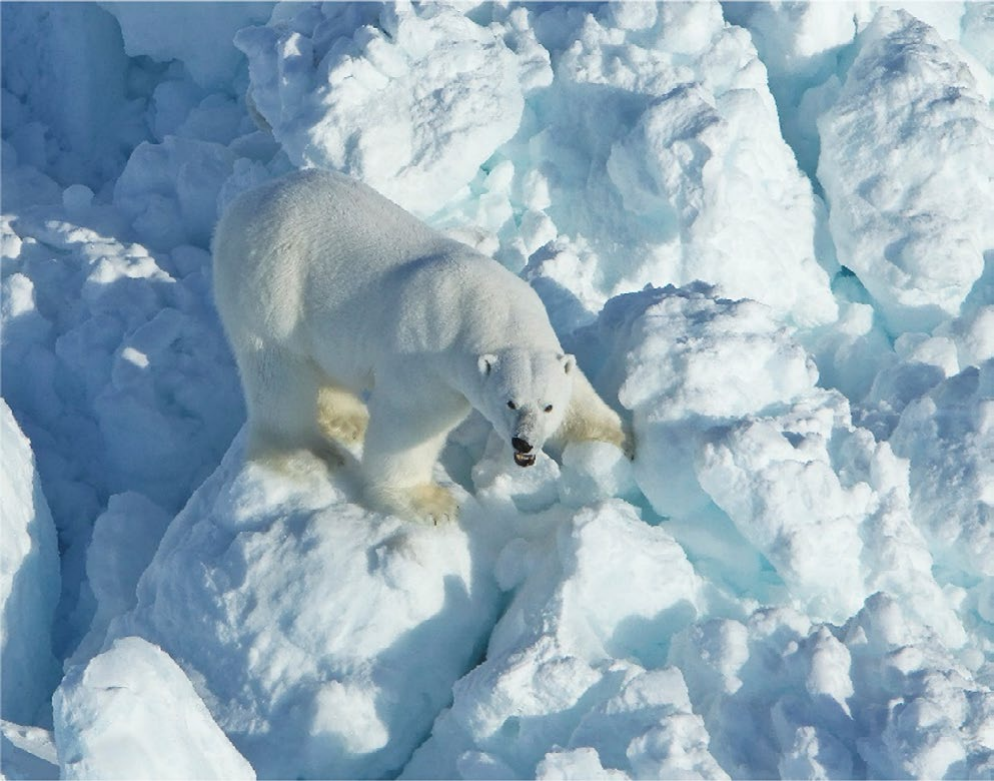
Male polar bear on rubble sea ice in the southern Beaufort Sea observed from a helicopter during mark–recapture operations in the spring of 2011. Photo credit: Mike Lockhart, US Geological Survey, public domain

We sampled bears on the sea ice (Figure 1) between longitudes 141.0° W and 158.5° W and as far as 103 km offshore during the spring (late March to mid‐May) of each year from 2001 to 2016 using a helicopter. Either bears were anesthetized with a drug‐filled dart and physically captured (2001–2016) or a tissue sample was collected without capture using a biopsy dart (2011–2013, yearlings and older; Pagano et al., 2014). Bears captured for the first time were given a unique identifying number tattooed on the buccal cavity side of the left and right upper lips and a numbered plastic ear tag in both ear pinnae so they could be identified upon recapture in subsequent years. Beginning in 2013, a microchip with a unique numeric code was injected subcutaneously behind one ear pinna. In most years, some captured bears were released with telemetry devices (deployed via collars, ear tags, or glued to fur) that allowed their locations to be monitored by satellite. Prior to release, captured bears were temporarily marked with a unique number painted on their backs to permit visual identification from the air during the remainder of the spring and prevent unintended recapture; biopsy darts similarly marked bears with paint to prevent recapture. Bears from which a biopsy sample was collected were later identified using genetic information (e.g., Paetkau, 2003), and many bears that were physically captured were also identified genetically.

Aircraft flight paths while searching for bears were recorded using GPS instruments. Because flight paths were not collected using a consistent time increment in all years, we used the R package “crawl” (Johnson & London, 2018; Johnson et al., 2008) to standardize all flight paths so that a location was estimated every 30 s. The minimum distance from each estimated location to the mainland coast was computed. Changes in activity (searching for bears, following tracks, tracking a VHF signal, traveling, etc.) during flights were recorded, which we used to identify the segments of each flight track during which the crew was actively searching for bears. However, changes in activity were not always recorded. If the activity data implied that the crew was searching for bears but the distance between successive location estimates was less than 10 m or greater than 2083 m, the speed was deemed incompatible with search activity and the leading location record was removed. Similarly, if a bear was captured during a segment in which the activity data implied the crew was not searching for bears, the preceding activity record was modified.

Tracking data for telemetry‐instrumented bears were standardized using methods similar to those used for the flight tracks, but with a 6‐hr increment between location estimates. The 12:00‐hr (UTC) location estimate for each bear in each day was retained for analysis, and the minimum distance from each of those estimated locations to the mainland coast was computed.

### 2.2 Spatial state design

**FIGURE 2:**
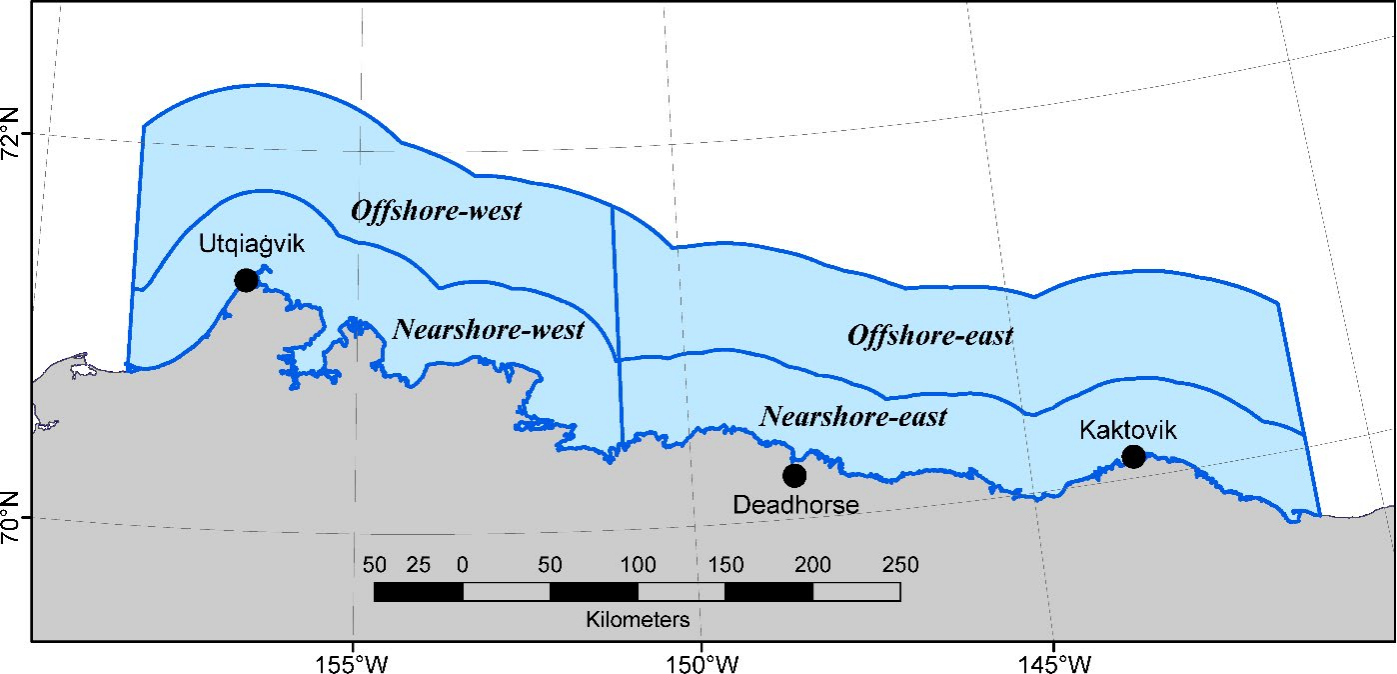
Southern Beaufort Sea study area off the northern coast of Alaska showing the four spatial states in which mark–recapture data were collected: (1) Nearshore‐west, (2) Nearshore‐east, (3) Offshore‐west, and (4) Offshore‐east. The region outside of those four states constituted a fifth state, Elsewhere

All investigators that have analyzed SBS mark–recapture data have recognized that heterogeneity in recapture probabilities, which can bias estimates of survival and abundance if not incorporated into model structure (Abadi et al., 2013; Carothers, 1979), could originate from spatial processes such as bear space use and the distribution of capture effort, and have therefore used various systems of spatial covariates to model heterogeneity (Amstrup et al., 2001; Bromaghin et al., 2015; Regehr et al., 2010). There are potential drawbacks with some spatial covariates, such as inaccuracies assigning bears to a region based on their first or average capture locations. We approached this problem by dividing the study area into spatial states that we suspected had different capture probabilities based on our understanding of bear habitat utilization and our field logistics. We defined a nearshore zone as the region within 43.246 km of the mainland coast, which was the 67th quantile of the distances from the coast of aircraft locations while actively searching for bears. This region would tend to contain the interface between land‐fast and pack ice and have high‐value habitat (Durner et al., 2009, 2019). An offshore zone extended from the nearshore zone out to 104.606 km, the 99.5th quantile of aircraft distances, which encompassed all capture and biopsy locations. The offshore zone was therefore larger than the nearshore zone, but received less capture effort over the entire study period and might therefore be expected to have lower capture probabilities. The difference in recapture probabilities between bears in the eastern and western regions of the Alaska SBS (e.g., Bromaghin et al., 2015) motivated a longitudinal division at −151.0° W, a locale in which relatively few captures have occurred historically. Consequently, the area encompassing all mark–recapture observations was partitioned into four spatial states from 141.0° W to 158.5° W and offshore to 104.606 km: State 1: Nearshore‐west, State 2: Nearshore‐east, State 3: Offshore‐west, and State 4: Offshore‐east (Figure 2). The region outside of these four spatial states comprised a fifth spatial state, State 5: Elsewhere, to accommodate bears moving westward into the Chukchi Sea subpopulation range, eastward into the North Beaufort Sea subpopulation range, or farther offshore into the Arctic Basin. We expected this system of spatial states to provide a realistic and computationally effective means of modeling heterogeneity in recapture probabilities.

Each bear that contributed data in a particular year was assigned to a single spatial state in that year. Each mark–recapture observation was assigned to the state in which it occurred. Each telemetry‐instrumented bear was also assigned to a single state each year it contributed data. The locations of telemetered bears were first censored, retaining the locations occurring between the first and last mark–recapture observation each spring to maximize the temporal coincidence of the two data sources. If the majority of a bear's estimated locations were in the Elsewhere state, it was assigned to that state; otherwise, it was assigned to the states 1–4 in which the greatest number of its locations occurred. We computed the total distance flown within each spatial state while searching for bears each year for use in modeling recapture probabilities. A few flights in some years had missing flight tracks, so we increased the distances flown within states in those years by the proportion of flight time without tracks to approximate the missing data. State assignments, data specific to individual bears, and the distances flown within each state in each year were compiled and released by Bromaghin et al. (2020).

### 2.3 Mark–recapture model

We used a Bayesian implementation of multistate Cormack–Jolly–Seber (CJS) models (Lebreton & Pradel, 2002) to estimate survival, state‐transition, and recapture probabilities, along with related model parameters that were not of direct interest. We kept the model space small by only considering four models with high degrees of structural flexibility to fit whatever patterns existed within the data, recognizing that prior analyses of SBS mark–recapture data have demonstrated that models with both age‐class and temporal structure are required to adequately approximate the data (Amstrup et al., 2001; Bromaghin et al., 2015; Regehr et al., 2010). The four models were formed as the pairwise combinations of two submodels for survival probabilities and two submodels for recapture probabilities. All four models used the same submodel for state‐transition probabilities.

Both survival submodels had a parameter for sex and parameters for each of seven age classes: Age0 (cubs of the year), Age1 (yearlings), Age2 (first year independent), Ages3–4 (subadults), Ages5–19 (adults), Ages20+ (old adults), and bears whose age was unknown. These age classes are similar to those used by others, with the most notable difference being the use of the Age2 class, which was motivated by our hypothesis that subadult survival might be lower in the first year a young bear is independent from its mother. Time (year) was either additive [Sex + Age + Time] or partially interacted with age [Sex + Age * Time]. Let $\phi_{\mathrm{ijk}}$ denote the probability a polar bear of sex *i* ∈ {F, M} in age class *j* ∈ {0, 1, 2, 3, 5, 20, U} survives from year *k* to *k* + 1, *k* ∈ {2001:2015}; note that the youngest age within an age class is used to index the parameter; for example, *j* = 5 indexes the Ages5–19 age class.

The time‐additive survival submodel [Sex + Age + Time] had survival probabilities for all sex and age classes that varied through time but were parallel on the logit scale. The probabilities were expressed relative to males in the Ages5–19 age class, so there was a parameter for each year ($\alpha_{T5k}$) that was shared by all sex and age classes, an additional parameter for each age class except the Ages5–19 and Unknown classes ($\alpha_{\mathrm{Aj}},j\left\{0,1,2,3,20\right\}$), and a parameter for sex ($\alpha_{F}$), that is,

$$\phi_{M0k}={\left\{1+e^{\alpha_{A0}+\alpha_{A1}+\alpha_{T5k}}\right\}}^{‐1},$$

$$\phi_{F0k}={\left\{1+e^{\alpha_{F}+\alpha_{A0}+\alpha_{A1}+\alpha_{T5k}}\right\}}^{‐1},$$

$$\phi_{M1k}={\left\{1+e^{\alpha_{A1}+\alpha_{T5k}}\right\}}^{‐1},$$

$$\phi_{F1k}={\left\{1+e^{\alpha_{F}+\alpha_{A1}+\alpha_{T5k}}\right\}}^{‐1},$$

$$\phi_{M2k}={\left\{1+e^{\alpha_{A2}+\alpha_{A3}+\alpha_{T5k}}\right\}}^{‐1},$$

$$\phi_{F2k}={\left\{1+e^{\alpha_{F}+\alpha_{A2}+\alpha_{A3}+\alpha_{T5k}}\right\}}^{‐1},$$

$$\phi_{M3k}={\left\{1+e^{\alpha_{A3}+\alpha_{T5k}}\right\}}^{‐1},$$

$$\phi_{F3k}={\left\{1+e^{\alpha_{F}+\alpha_{A3}+\alpha_{T5k}}\right\}}^{‐1},$$

$$\phi_{M5k}={\left\{1+e^{\alpha_{T5k}}\right\}}^{‐1},$$

$$\phi_{F5k}={\left\{1+e^{\alpha_{F}+\alpha_{T5k}}\right\}}^{‐1},$$

$$\phi_{M20k}={\left\{1+e^{\alpha_{A20}+\alpha_{T5k}}\right\}}^{‐1},\text{and}$$

$$\phi_{F20k}={\left\{1+e^{\alpha_{F}+\alpha_{A20}+\alpha_{T5k}}\right\}}^{‐1}.$$

This parametric structure for the survival probabilities, in combination with the prior distributions.

$$\alpha_{F}∼\text{uniform}\left(‐5,5\right),$$

$$\alpha_{\mathrm{Aj}}∼\text{uniform}\left(0,5\right),j\left\{0,1,2,3,20\right\},\;\text{and}$$

$$\alpha_{T5k}∼\text{uniform}\left(‐5,5\right),\;k\left\{2001:2015\right\},$$

imposed the following constraints on survival:

$$\phi_{i0k}\leq \phi_{i1k}\leq \phi_{i5k},$$

$$\phi_{i2k}\leq \phi_{i3k}\leq \phi_{i5k},\;\text{and}$$

$$\phi_{i20k}\leq \phi_{i5k},\;i\;\left\{F,\;M\right\}.$$

These stabilizing constraints derive logically from the definition of the age classes and are consistent with biological expectations and prior estimates of survival for SBS polar bears (e.g., Bromaghin et al., 2015).

The time‐interactive survival submodel [Sex + Age * Time] was structured similarly, except that three independent sets of time parameters were used, one set each for the Age0 and Age2 age classes, and the third set for the other age classes combined. The increased temporal complexity of this model was implemented to allow the survival probabilities of the potentially most vulnerable age classes to vary independently through time (Bromaghin et al., 2015). In this survival submodel, survival probabilities for the Age0 and Age2 age classes were defined as:

$$\phi_{M0k}={\left\{1+e^{\alpha_{T0k}+\alpha_{A1}+\alpha_{T5k}}\right\}}^{‐1},$$

$$\phi_{F0k}={\left\{1+e^{\alpha_{F}+\alpha_{T0k}+\alpha_{A1}+\alpha_{T5k}}\right\}}^{‐1},$$

$$\phi_{M2k}={\left\{1+e^{\alpha_{T2k}+\alpha_{A3}+\alpha_{T5k}}\right\}}^{‐1},\;\text{and}$$

$$\phi_{F2k}={\left\{1+e^{\alpha_{F}+\alpha_{T2k}+\alpha_{A3}+\alpha_{T5k}}\right\}}^{‐1},$$

where

$$\alpha_{T0k}∼\text{uniform}\left(0,5\right)\text{and}$$

$$\alpha_{T2k}∼\text{uniform}\left(0,5\right),k\left\{2001:2015\right\}.$$

Survival probabilities for the other age classes were unchanged from the time‐additive submodel.

For both submodels, survival probabilities for bears whose age was unknown (primarily biopsy‐darted individuals) were modeled as a weighted average of the survival probabilities for the Ages3–4, Ages5–19, and Ages20+ age classes;

$$\phi_{\mathrm{MUk}}=\alpha_{W1}\phi_{M3k}+\left(1‐\alpha_{W1}\right)\alpha_{W2}\phi_{M5k}+\left(1‐\alpha_{W1}\right)\left(1‐\alpha_{W2}\right)\phi_{M20k}\;\text{and}$$

$$\phi_{\mathrm{FUk}}=\alpha_{W1}\phi_{F3k}+\left(1‐\alpha_{W1}\right)\alpha_{W2}\phi_{F5k}+\left(1‐\alpha_{W1}\right)\left(1‐\alpha_{W2}\right)\phi_{F20k},$$

where

$$\alpha_{\mathrm{Wi}}∼\text{uniform}\left(0,1\right),i\left\{1:2\right\}.$$

The weights $\alpha_{W1}$, $\left(1‐\alpha_{W1}\right)\alpha_{W2}$, and $\left(1‐\alpha_{W1}\right)\left(1‐\alpha_{W2}\right)$ are positive, sum to 1, and were viewed as uninteresting parameters whose purpose was simply to approximate the survival of bears with unknown age.

State‐transition probabilities were first‐order Markovian (depending only on the state currently occupied), equal for age and sex classes, and constant through time. The probability of transitioning from state m in year k to state n in year *k* + 1, $\tau_{\mathrm{mn}}$, was parameterized as the product of probabilities with uniform prior distributions:

$$\tau_{m1}=\upsilon_{m1},$$

$$\tau_{m2}=\left(1‐\upsilon_{m1}\right)\upsilon_{m2},$$

$$\tau_{m3}=\left(1‐\upsilon_{m1}\right)\left(1‐\upsilon_{m2}\right)\upsilon_{m3},$$

$$\tau_{m4}=\left(1‐\upsilon_{m1}\right)\left(1‐\upsilon_{m2}\right)\left(1‐\upsilon_{m3}\right)\upsilon_{m4},\;\text{and}$$

$$\tau_{m5}=\left(1‐\upsilon_{m1}\right)\left(1‐\upsilon_{m2}\right)\left(1‐\upsilon_{m3}\right)\left(1‐\upsilon_{m4}\right),$$

where

$$\upsilon_{\mathrm{mn}}∼\text{uniform}\left(0,1\right),m\left\{1:5\right\},\;n\left\{1:4\right\}.$$

This parameterization bounded the transition probabilities in the interval (0, 1) and constrained the probabilities associated with any state of origin to sum to 1, that is, $\sum_{n}\upsilon_{\mathrm{mn}}=1$. We did not explore generalizations of the transition probabilities because of the large number of additional parameters that would have been required.

One recapture probability submodel incorporated parameters for state and time [State + Time]. Let $\rho_{\mathrm{ik}}$ denote the probability of recapture for a bear located in spatial state *i* in year *k*, that is,

$$\rho_{1k}={\left\{1+e^{\beta_{1k}}\right\}}^{‐1},$$

$$\rho_{2k}={\left\{1+e^{\beta_{1k}+\beta_{2}}\right\}}^{‐1},$$

$$\rho_{3k}={\left\{1+e^{\beta_{1k}+\beta_{3}}\right\}}^{‐1},\;\text{and}$$

$$\rho_{4k}={\left\{1+e^{\beta_{1k}+\beta_{2}+\beta_{4}}\right\}}^{‐1},$$

where

$$\beta_{1k}∼\text{uniform}\left(‐5,5\right),k\left\{2002:2016\right\},$$

$$\beta_{2}∼\text{uniform}\left(‐5,5\right),\;\text{and}$$

$$\beta_{i}∼\text{uniform}\left(0,5\right),i\left\{3,4\right\}.$$

This parametric structure produces recapture probabilities $\rho_{\mathrm{ik}}$ that are parallel through time on the logit scale, and the prior distributions for $\beta_{3}$ and $\beta_{4}$ impose the constraints $\rho_{1k}\geq \rho_{3k}$ and $\rho_{2k}\geq \rho_{4k}$, which are consistent with the delineation between the nearshore and offshore states being based on the distribution of search effort and with the majority of the capture effort occurring in the nearshore states, as described earlier.

The second submodel for recapture probabilities was structured similarly, but included parameters for the distance flown in state m during year *k*, $d_{\mathrm{mk}}$,

$$\rho_{1k}=\omega {\left\{1+e^{\beta_{1k}+\theta_{1}d_{1k}}\right\}}^{‐1},$$

$$\rho_{2k}=\omega {\left\{1+e^{\beta_{1k}+\beta_{2}+\theta_{2}d_{2k}}\right\}}^{‐1},$$

$$\rho_{3k}=\omega {\left\{1+e^{\beta_{1k}+\beta_{3}+\theta_{3}d_{3k}}\right\}}^{‐1},\text{and}$$

$$\rho_{4k}=\omega {\left\{1+e^{\beta_{1k}+\beta_{2}+\beta_{4}+\theta_{4}d_{4k}}\right\}}^{‐1},$$

where

$$\omega ∼\text{uniform}\left(0.01,1\right)\;\text{and}$$

$$\theta_{m}∼\text{uniform}\left(‐50,0\right),m\left\{1:4\right\},\;\text{and}$$

$$k\in \left\{2002:2016\right\}.$$

The likelihood function had components for the capture histories of mark–recapture bears and the interannual transition of telemetry‐instrumented bears between states. CJS analyses condition on the first observation of a marked bear and then model its subsequent capture history to the end of the study period. Given the initial observation of a bear of sex *i* and age class *j* in state m during year *k*, it survived to year *k* + 1 and occupied state *n* with probability $\left\{\phi_{\mathrm{ijk}}\tau_{\mathrm{mn}}\right\}$, *n* ∈ {1:5}, or died and moved into a sixth nonspatial “dead” state with probability $\left\{1‐\phi_{\mathrm{ijk}}\right\}$. Note that state occupancy was a partially observed latent variable because its values were known in years that bears were observed and internally estimated in other years; that is, the combination of survival and state transition defines a state process that is not fully observable. Given that a bear was alive and occupying state *n* in year *k*, the outcome of the observation process (observed or unobserved) was a Bernoulli random variable with probability $\left\{\rho_{\mathrm{nk}}\right\}$. A bear that was alive and occupying State 5: Elsewhere or dead (occupying State 6) was unobserved with probability 1. We also conditioned on the state occupied by telemetry‐instrumented bears during the first spring they contributed location data and modeled their state occupancy forward in time until the last year they contributed data. They were known to be alive during this period, so their survival was not modeled. Given that an instrumented bear was in state m during year *k*, it occupies state *n* in year *k* + 1 with probability $\left\{\tau_{\mathrm{mn}}\right\}$, *n* ∈ {1:5}. Consequently, instrumented bears were only informative with respect to state‐transition probabilities. Treating the data from instrumented bears separately in this way exploited their most important information, i.e. high‐quality locations, and avoided introducing a source of heterogeneity in recapture probabilities because those bears were generally targeted for recapture if their locations were known and within range. Note that observations of a bear that was telemetry‐instrumented at some point in its lifetime were included in the mark–recapture data if its capture occurred during the normal course of mark–recapture activities and it was not targeted using a priori knowledge of its location.

Estimates of abundance were derived from estimated probabilities of occupancy in states 1–4 and recapture given occupancy using a Horvitz–Thompson estimator (Horvitz & Thompson, 1952; McDonald & Amstrup, 2001). The number of bears occupying states 1–4 in year *k*, $N_{k}^{*}$, was estimated as.

$${\hat{N}}_{k}^{*}=\sum_{m=1}^{4}C_{\mathrm{mk}}/{\hat{\rho }}_{\mathrm{mk}},$$

where $C_{\mathrm{mk}}$ is the number of bears captured in state *m* in year *k*, *k* ∈ {2002:2015}. The total number of bears alive, $N_{k}$, was estimated by dividing ${\hat{N}}_{k}^{*}$ by the proportion of bears estimated to be alive in year k (occupying states 1–5) that were in states 1–4,

$${\hat{N}}_{k}={\hat{N}}_{k}^{*}{\left(\frac{\text{Number of mark}‐\text{recapture bears in states}\;1‐4\;\text{in year}\;k}{\text{Number of mark}‐\text{recapture bears in states}\;1‐5\;\text{in year}\;k}\right)}^{‐1}.$$

Parameters were estimated using Bayesian methods in R version 3.6.3 (R Core Team, 2020), using jagsUI (Kellner, 2019) to interface with rjags (Plummer, 2019). For each model, five Markov chains were initialized with random starting points and a burn‐in of 200,000 iterations and then continued for an additional 100,000 iterations, storing every 25th iteration and thereby producing samples of 20,000 from posterior distributions. Convergence was assessed using the Gelman–Rubin convergence diagnostic *R_c_*, with values less than 1.1 indicative of convergence (Gelman & Rubin, 1992), and by examining Markov chain trace plots for signs of adequate mixing. Models were compared using the deviance information criterion (DIC, e.g., Spiegelhalter et al., 2014). Goodness of fit was evaluated using the Bayesian p‐values computed from 20,000 Freeman–Tukey statistics comparing the fit between the observed data and model‐conditioned expected values with that of model‐conditioned simulated data and expected values (Kéry & Schaub, 2012, section 7.10.2).

We performed a rough check on the consistency between survival and abundance estimates by comparing the trend in abundance estimates with the trend in population projections simulated using methods similar to those of Bromaghin et al. (2015, appendix E). An initial population consisting of a fixed number of family groups of randomized composition was established in Year 1. That initial population was projected 14 years forward in time using a set of samples from the posterior distributions of the CJS survival probabilities and a simple data‐based method of simulating reproduction. A projection was completed with each of the 20,000 sets of samples from the survival probability posterior distributions. The appendix contains additional details on these projections.

## 3 RESULTS

The mark–recapture data were comprised of 1,224 observations of 868 individual bears, including 93 genetic identifications from biopsy tissue samples and 356 recaptures (Table 1). The number of bears observed each year ranged from 124 in 2004 to 21 in 2016 and averaged 77 (Table 1). The number of observations in most combinations of age class, sex, and year was greater than 0 (Table S1), which helps achieve identifiability of the survival probabilities together with their parallel structure. Most bears were only observed once (657, 75.7%) though one bear was observed in 8 years (Table S2), including the first and last years of the study. A total of 122 telemetered bears provided state occupancy data during at least two years.

**TABLE 1:** Number of Southern Beaufort Sea polar bear mark–recapture observations in Alaska from 2001 to 2016 (C) and the number of those individuals that were recaptures (R), by observation type and year

Year	Capture		Biopsy		Total	
	C	R	C	R	C	R
2001	63	0	0	0	63	0
2002	78	8	0	0	78	8
2003	92	9	0	0	92	9
2004	124	22	0	0	124	22
2005	80	22	0	0	80	22
2006	85	31	0	0	85	31
2007	76	22	0	0	76	22
2008	78	25	0	0	78	25
2009	92	36	0	0	92	36
2010	65	29	0	0	65	29
2011	61	20	39	18	100	38
2012	74	30	24	12	98	42
2013	56	20	30	13	86	33
2014	48	20	0	0	48	20
2015	38	12	0	0	38	12
2016	21	7	0	0	21	7

The combined mark–recapture and telemetry data set contained a total of 1,538 observations among the five spatial states, 361 in State 1: Nearshore‐west, 604 in State 2: Nearshore‐east, 83 in State 3: Offshore‐west, 345 in State 4: Offshore‐east, and 145 in State 5: Elsewhere (Table S3). Of that total, there were 312 instances (20.0%) in which bears were observed the following year so that state transitions were known without error.

The four candidate models ran on a Windows 10 computer with Intel Zeon 10‐core 2.20 GHz processors and 256 GB of RAM; completion times ranged from 87 to 153 hr and averaged 128 hr. The model with additive time structure in both the survival and recapture probability submodels provided the most parsimonious fit to the data (Table 2) and was selected as the preferred model. Because both survival and recapture probabilities varied through time, those parameters were confounded in the last year of the study, and so 2015 survival probabilities, 2016 recapture probabilities, and 2016 abundance could not be estimated. The maximum value of the Gelman–Rubin convergence diagnostic *R_c_* was 1.066 among all parameters and 1.006 when the confounded parameters in the last year were excluded. The Bayesian *p*‐values were all near 0.5 (Table 2), suggesting that the models provided adequate fit to the observed data. The Markov chains appeared well mixed in all trace plots.

**TABLE 2:** Comparison of the multistate Cormack–Jolly–Seber models used to estimate survival and recapture probabilities of Alaska Southern Beaufort Sea polar bears, 2001–2016, showing the structure of the survival and recapture probability submodels, the number of parameters in the model (NP), the Bayesian *p*‐value summarizing goodness of fit (*p*_B_) to the data, the deviance information criterion (DIC) measure used to compare models, and the difference between each model's DIC and that of the smallest DIC

Survival	Recapture	NP	*p*_B_	DIC	ΔDIC
Sex + Age + Time	State + Time	61	0.504	5,553.0	0
Sex + Age * Time	State + Distance	80	0.448	5,565.4	12.4
Sex + Age + Time	State + Distance	52	0.438	5,571.5	18.5
Sex + Age * Time	State + Time	89	0.500	5,611.4	58.1

**FIGURE 3:**
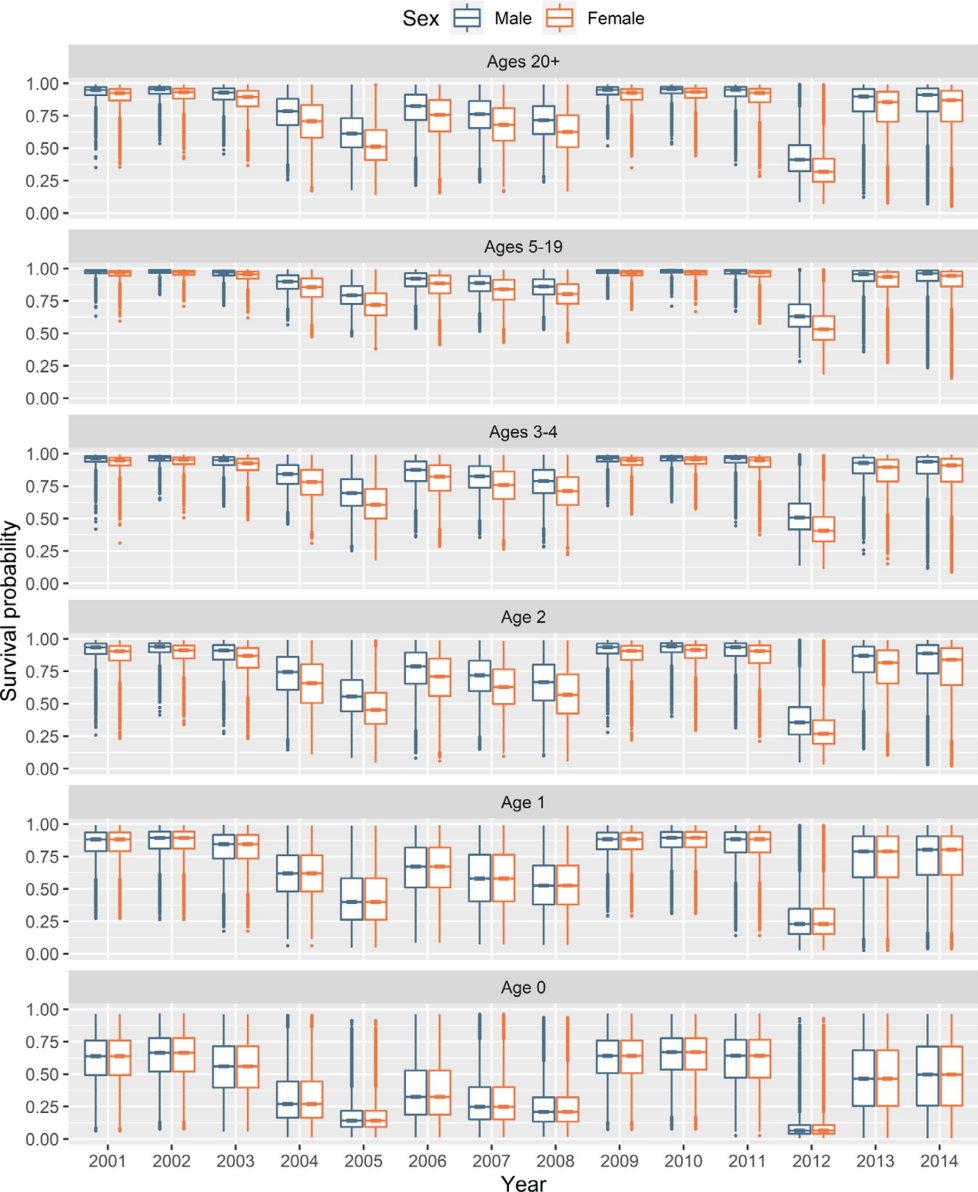
Annual survival probability estimates for polar bears of the Alaska portion of the southern Beaufort Sea. Each boxplot is based on a sample of size 20,000 drawn from the survival probability posterior distribution for a specific combination of sex, age class, and year. The line in the middle of each “box” is the median, the lower and upper extents of each box are the 25th and 75th quartiles, and the “whiskers” extend to the most extreme values that are no more than 1.5 times the box width below or above the 25th and 75th quartiles, respectively, with any more extreme values plotted individually. Survival probabilities pertain to a one‐year period beginning in the spring of the year with which they are labeled

Annual survival probability estimates from the preferred model had distinct periods of low and high survival through time (Figure 3, Tables S4–S10). In general, survival probabilities were high from 2001 to 2003, lower from 2004 to 2008, and then high again from 2009 through 2015, with the notable exception of 2012. This temporal pattern in survival probabilities was similar to the patterns reported by other investigators from 2001 through 2005 (Regehr et al., 2010) and 2009 (Bromaghin et al., 2015). The relative magnitudes of estimated survival probabilities among the age classes, some of which were imposed as described above, were Age0 < Age1 < Age2 < Ages20+ < Ages3–4 < Ages5–19. Female survival was slightly less than male survival, as previously reported for the SBS subpopulation (Bromaghin et al., 2015; Regehr et al., 2010). The usual tension between survival and recapture probabilities is reflected in the long, skewed tails of the posterior densities for survival (Figure 3), but the mass of the posterior densities is concentrated about the point estimates.

**FIGURE 4:**
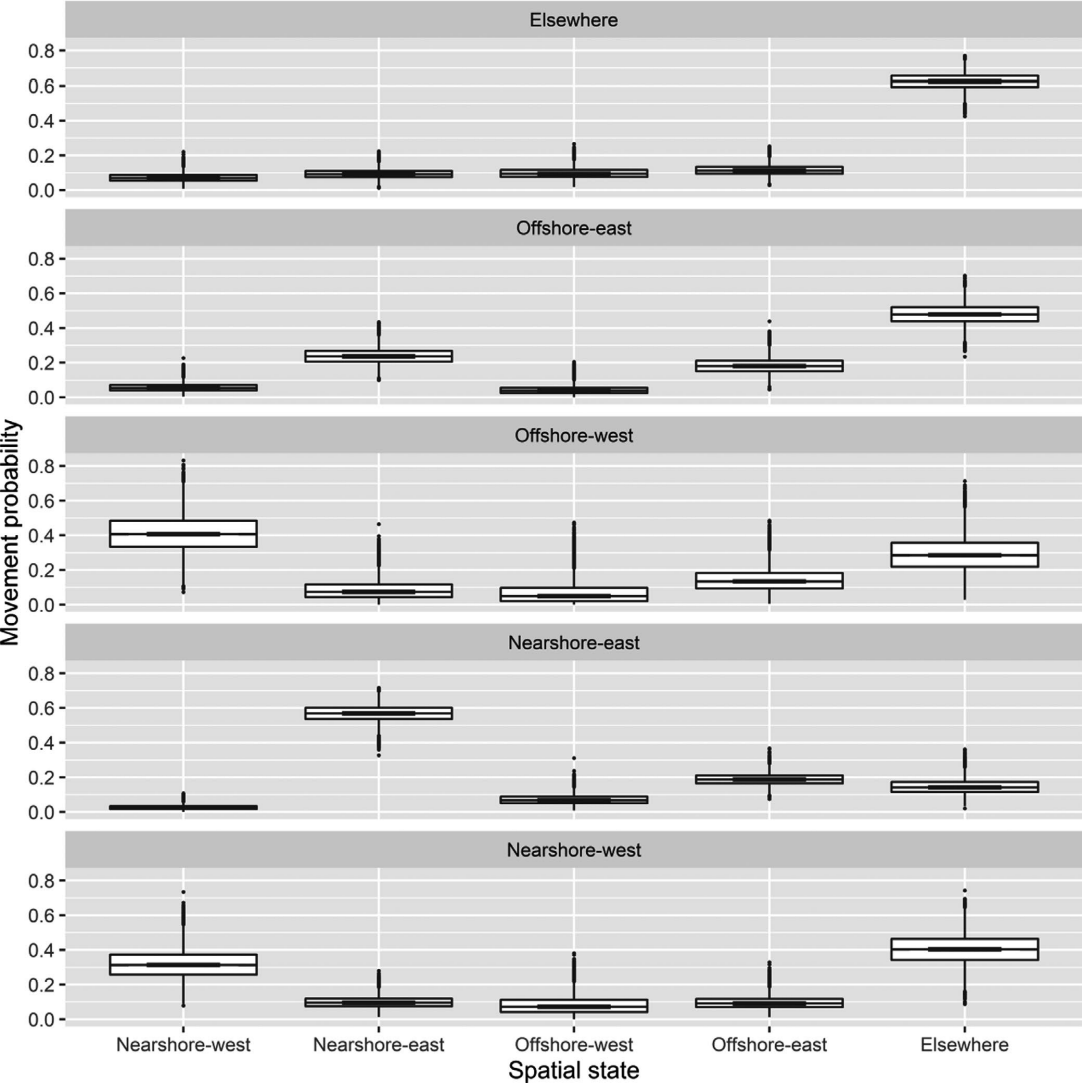
Estimated probabilities of a polar bear from the Alaska portion of the southern Beaufort Sea remaining in a spatial state or transitioning to another spatial state between capture occasions. States of origin are given in panels, with states of destination along the horizontal axis. Each boxplot is based on a sample of size 20,000 drawn from the posterior distribution of state‐transition probabilities for each combination of states of origin and destination. The line in the middle of each “box” is the median, the lower and upper extents of each box are the 25th and 75th quartiles, and the “whiskers” extend to the most extreme values that are no more than 1.5 times the box width below or above the 25th and 75th quartiles, respectively, with any more extreme values plotted individually

**FIGURE 5:**
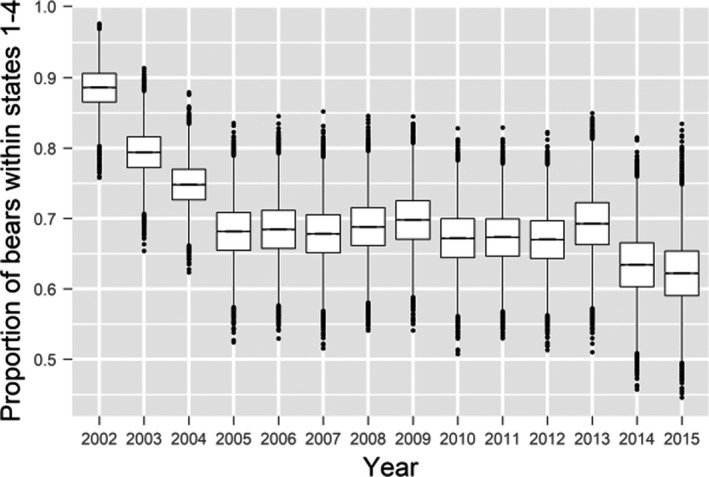
The proportion of marked bears known or modeled to be alive (occupying states 1–5) that were known or modeled to occupy states 1–4, by year. Each boxplot is based on a sample of 20,000 drawn from the posterior distribution of the proportion in each year. The line in the middle of each “box” is the median, the lower and upper extents of each box are the 25th and 75th quartiles, and the “whiskers” extend to the most extreme values that are no more than 1.5 times the box width below or above the 25th and 75th quartiles, respectively, with any more extreme values plotted individually. High values in the first years of the study period were caused by spatially limited sampling in 2001 and incomplete mixing of marked and unmarked bears between states 1–4 and state 5

The state‐transition probabilities were estimated with high precision, likely because of the large proportion of transitions that were observed (observations of the same bear in consecutive years), and showed strong evidence of Markovian movement between spatial states (Figure 4, Table S11), as was preliminarily reported by Bromaghin et al. (2015). Bears were most likely to remain in the Nearshore‐west, Nearshore‐east, and Elsewhere states. Fidelity to the eastern and western states was also apparent; for example, bears in the Offshore‐east state had higher probabilities of remaining in that state or moving to the Nearshore‐east state than moving to either of the western states. In a typical year, approximately two thirds of the population was estimated to be within states 1–4 during the spring field season (Figure 5, Table S12).

**FIGURE 6:**
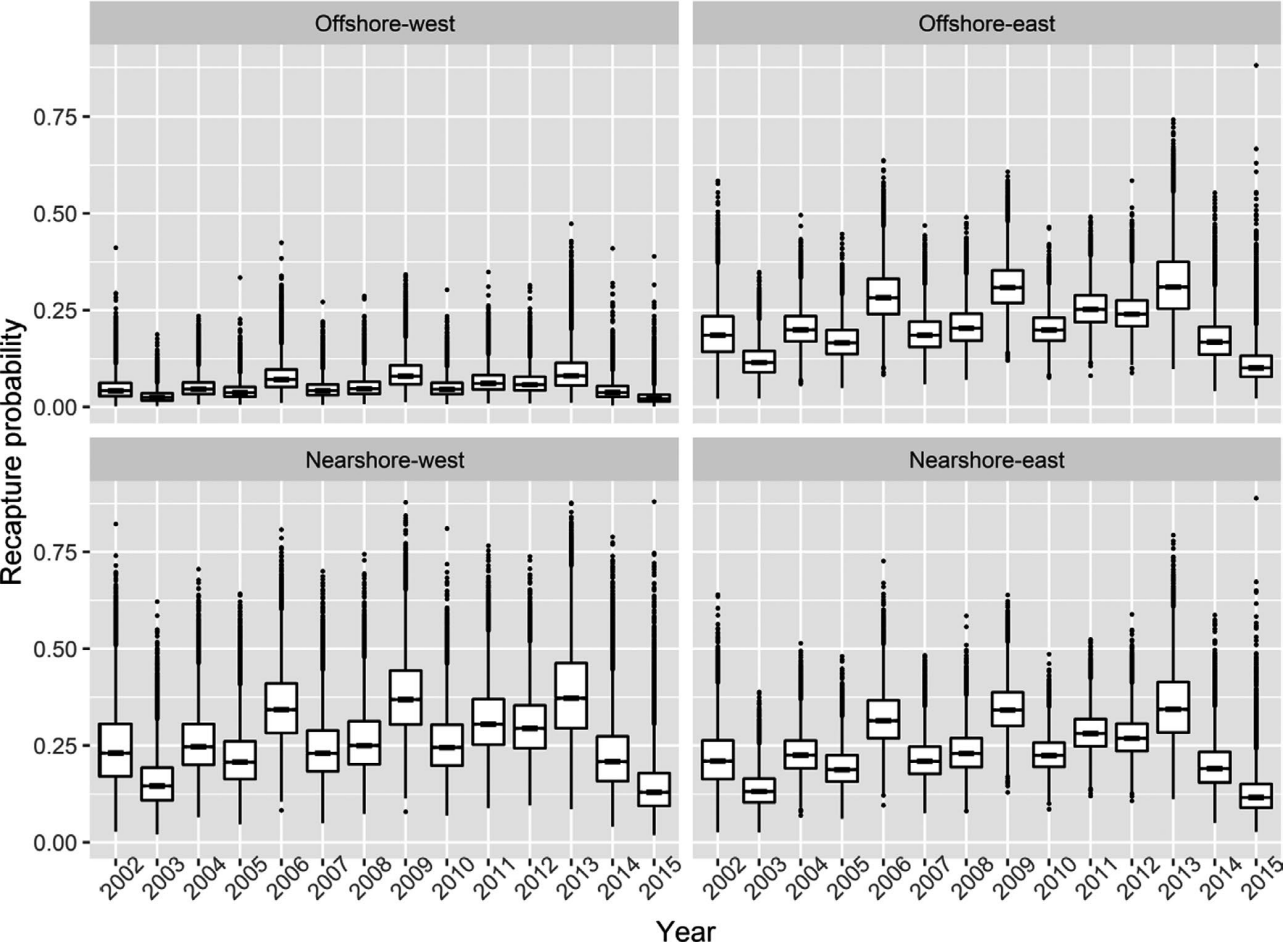
Estimated recapture probabilities for polar bears of the Alaska portion of the southern Beaufort Sea by spatial state and year. Each boxplot is based on a sample of 20,000 drawn from the recapture probability posterior distribution for a specific combination of state and year. The line in the middle of each “box” is the median, the lower and upper extents of each box are the 25th and 75th quartiles, and the “whiskers” extend to the most extreme values that are no more than 1.5 times the box width below or above the 25th and 75th quartiles, respectively, with any more extreme values plotted individually

Recapture probability estimates did not display any obvious patterns through time, but rather seemed to fluctuate around an overall mean, though estimates were lower than average in 2014 and 2015 (Figure 6, Table S13). Bears occupying offshore states had lower recapture probabilities than bears in nearshore states, consistent with the design of the states and the constraints imposed by prior distributions previously described. Recapture probabilities in State 3 (Offshore‐west) were notably lower than in states 1, 2, and 4, whose recapture probabilities were more similar in comparison.

**FIGURE 7:**
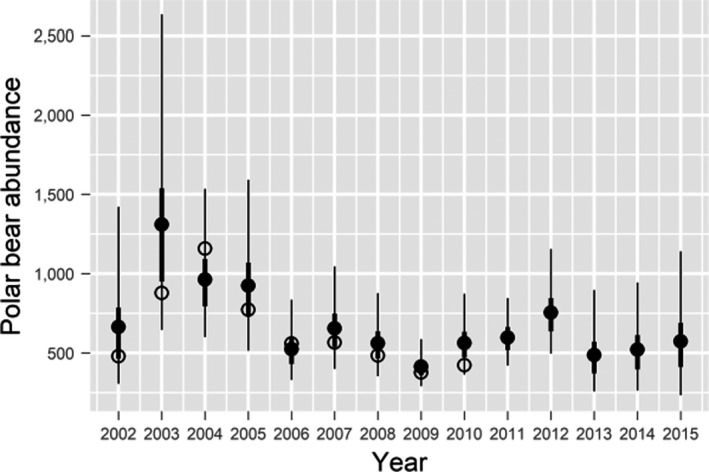
Estimates of the abundance of polar bears in the Alaska portion of the southern Beaufort Sea, based on a sample of 20,000 drawn from the posterior distribution of abundance in each year. Filled circles are the average estimates, and the thick and thin lines represent the 50% and 95% credible intervals, respectively. Open circles are the point estimates reported by Bromaghin et al. (2015). Note that the estimate for 2002 was known to be negatively biased and abundance was not thought to have meaningfully increased from 2002 to 2003

The temporal pattern in abundance estimates was consistent with the estimated survival probabilities (Figure 7, Table S14). Abundance declined substantially from 2003 to 2006, years in which survival was estimated to be low (Figure 3). Abundance fluctuated less dramatically at lower levels and in accord with survival from 2006 to 2015, reaching a low in 2009; it would also be reasonable to characterize abundance as generally falling from 2003 to the low in 2009. Higher survival from 2009 to 2011 led to modest population growth that was reversed by unusually low survival in 2012. Estimated abundance in 2015, the last year we could obtain an estimate, was 573 (95% Bayesian credible interval [232, 1140]); note the typical increased uncertainty in the estimate for the last year. Given variation associated with the annual abundance estimates, subpopulation abundance from 2006 to 2015 could be characterized as below earlier levels but relatively stable, averaging 565 (average 95% BCI [340, 920]) bears over that period. The most notable difference between the temporal trends in the abundance estimates and population projections occurred from 2009 to 2012, when estimated survival rates were high and abundance estimates were increasing, but the projections were unexpectedly stable. The trends were otherwise similar, and the projection results overall did not raise any concerns regarding inconsistency between estimates of abundance and survival probabilities (Figure S2).

## 4 DISCUSSION

Survival probabilities during the study period appeared to be bimodally distributed (Figure 3). For example, the estimated survival of females in the Ages5–19 age class during the years 2001–2003, 2009–2011, and 2013–2014 ranged from 0.89 to 0.96 and averaged 0.94, while estimates in the other years ranged from 0.55 to 0.87 and averaged 0.77 (Figure 3), although point estimates for 2012 seem implausibly low (the average excluding 2012 is 0.81). Survival probabilities for adult polar bears of approximately 0.90 or higher are common throughout the Arctic (e.g., Amstrup & Durner, 1995; Lunn et al., 2016; Peacock et al., 2013; Regehr et al., 2018; Taylor et al., 2009; Wiig, 1998), but lower survival probabilities are rare. The only estimates of adult survival in other subpopulations substantially <0.90 that we are aware of were reported by Stirling et al. (2011) for the neighboring Northern Beaufort Sea (NBS) subpopulation, with estimates for subadult and adult males as low as 0.77 in 2005. However, it is important to note that most investigators have not had sufficient data to estimate a nonparametric survival probability for each year as we have done; many published estimates are temporally invariant, and most annual estimates are modeled as a function of covariates and so may partially mask the true degree of temporal variation in survival.

Bimodal survival probabilities through time might be expected when a specialized species inhabits an isolated environment undergoing rapid degradation. Throughout most of their range, including the SBS, polar bears have historically had relatively predictable access to prey provided by seasonal but interannually stable sea ice phenology. The high survival and long life of adult polar bears throughout the Arctic since the curtailment of overharvest are consistent with expectations for an apex predator in a stable environment with adequate resources. However, Arctic sea ice habitat can no longer be considered stable; sea ice loss is accelerating (Serreze & Strove, 2015) and new record lows are occurring more frequently (Kumar et al., 2020). Increasing environmental degradation and variability can be expected to periodically result in extreme conditions that some individuals or certain segments of a population may be unable to survive. As the availability and quality of Arctic sea ice continue to worsen, periods of low survival are expected to become more common (Atwood, Marcot, et al., 2016; Molnár et al., 2020).

The specific conditions that contributed to low survival in 2004 to 2008 and 2012 are not known with certainty and causes may vary among years, but several independent sources of information collectively suggest that SBS polar bears were nutritionally stressed in the mid‐2000s. Cherry et al. (2009) found that the proportion of eastern Beaufort Sea bears fasting in the spring, when bears would normally be acquiring mass, more than doubled from 1985–1986 to 2005–2006. Rode et al. (2018) reported substantial increases in springtime fasting between 1983–1999 and 2000–2016 in both the NBS and SBS subpopulations. In the eastern Beaufort Sea, nearly twice as many ringed seal pup kill sites were observed in 2007–2011 than in 2003–2006 (Pilfold et al., 2014), although relative search effort in the two periods was not reported. Stirling et al. (2008) summarized evidence of poor hunting success in 2003–2007 and reported an instance of an adult female being cannibalized. Similarly, Amstrup et al. (2006) reported three instances of polar bears stalking, killing, and consuming other bears. Rode et al. (2010) hypothesized that reduced nutritional intake was responsible for declining trends in physical stature and reproductive output from 1982 to 2006. These observations are not incontrovertible evidence of unusually low survival in the mid‐2000s, but the SBS subpopulation was clearly not healthy and thriving during that period. Although we are not aware of similar evidence of low survival in 2012, it was a peak year for alopecia among spring‐captured SBS polar bears (Atwood et al., 2015), multiparous female ringed seals in Amundsen Gulf (east of our study area) had the second lowest ovulation rate since 1992 (Harwood et al., 2020), and the lowest Arctic sea ice extent recorded to date occurred in September 2012 (Kwok, 2018).

Reduced nutritional intake could be caused by some combination of low prey abundance, a reduction in the nutritional quality of prey, and limited access to prey. Unfortunately, quantitative information on the abundance of ringed and bearded seals, the primary prey of polar bears in most of the Arctic (e.g., Thiemann et al., 2008), is limited in the Beaufort Sea. In Amundsen Gulf in the eastern Beaufort Sea, the percentage of pups in the ringed seal harvest (considered a measure of recruitment) was below 25% from 2003 to 2007 and 2012 to 2014, the proportion of multiparous females that were ovulating was extremely low in both 2005 and 2012 (the two years in which our estimated survival rates were the lowest), and the blubber depth of adult ringed seals in the harvest trended downward from 1992 to 2019 (Harwood et al., 2020). Although Amundsen Gulf is east of our study area, the ringed seal harvest statistics are correlated with the Arctic Oscillation (Harwood et al., 2020) and so may be informative with respect to ringed seal production over a larger region. The steadily lengthening summer ice‐free period (Stern & Laidre, 2016) may increasingly limit access to prey during the early summer when SBS bears have historically exploited the availability of juvenile seals to accumulate body mass reserves. The majority of SBS bears currently remain on the sea ice as it melts back into the Arctic basin in summer (Atwood, Peacock, et al., 2016), where their activity rates decline (Ware et al., 2017) and they are largely food‐deprived (Whiteman et al., 2018). In addition, thin annual ice is susceptible to deformation during winter storms and the resulting rafted and jumbled ice can present formidable barriers to seal lair penetration in the spring (Stirling et al., 2008). These and likely other factors may be limiting the nutritional intake of SBS polar bears at a time when environmental conditions are increasing their energy requirements (Pagano et al., 2018, 2020).

The vitality and abundance of SBS polar bears appear to have been in general decline since the late 1990s (e.g., Amstrup et al., 2001; Bromaghin et al., 2015; Rode et al., 2010, 2018), but that period has been interspersed with intervals of higher survival and relative population stability (Figures 3 and 6). There are several possible explanations for periods of stability or growth to be nested within an overall downward trend. SBS polar bears occupy an increasingly dynamic ecosystem (Graham et al., 2017; Kwok, 2018), and the complex interaction of all factors that influence survival is undoubtedly more favorable in some years than others, potentially dampening or arresting population decline. For example, increased energetic requirements stemming from sea ice decline (e.g., Durner et al., 2017; Pagano et al., 2020) could, in some years, be more than counterbalanced by the increased productivity of prey (e.g., Harwood et al., 2020; Nguyen et al., 2017). If population growth is limited by resource scarcity, a period of low survival could ease density dependence and enhance survival in subsequent years (e.g., Hixon et al., 2002). Similarly, if a period of unfavorable conditions selectively eliminated individuals with inherently low survival, surviving individuals have successfully coped with the challenge and should collectively display an elevated average survival rate. Finally, behavioral modification in response to unfavorable conditions could temporarily increase survival and slow population decline. For example, since the early 2000s, a growing proportion of the SBS subpopulation has moved to terrestrial habitats when sea ice disappears from the continental shelf in summer (Atwood, Peacock, et al., 2016). While on land, most polar bears scavenge the remains of subsistence‐harvested bowhead whales (*Balaena mysticetes*), which may help mitigate declines in body condition (McKinney et al., 2017) and improve survival for some individuals, although this unique resource is limited. It is conceivable that such mechanisms have collectively resulted in years during which the SBS subpopulation remained stable or experienced growth under contemporary conditions, though abundance is substantially below prior levels. Even so, long‐term survival is expected to be driven by the degree to which adequate access to prey and other critical life‐history needs (Atwood, Marcot, et al., 2016; Molnar et al., 2020) can be maintained in future decades during which Arctic warming is projected to continue (e.g., SIMIP Community, 2020).

The increase in abundance estimates from 2002 to 2003 (Figure 6) is thought to be an artifact of project initiation and polar bear movement patterns. The 2002 abundance estimate is negatively biased because no capture effort was based out of Utqiaġvik in 2001, so there was little capture effort in spatial states 1 and 3. Capture effort was spread throughout spatial states 1–4 in 2002, so the first admissible abundance estimate is for 2003. In addition, Bromaghin et al. (2015) presented preliminary evidence that the probability a bear is within the region in which captures occur in any particular year is dependent on its location the preceding year, and in fact, we have modeled state‐transition probabilities as Markovian. Markovian movement with incomplete spatial distribution of marks implies that CJS abundance estimates will have some bias at the beginning of a study, but that bias will decrease with time as additional bears are marked and previously marked bears become more thoroughly mixed with unmarked bears (Figure 7). Judging from the relatively high estimates of survival in 2001 and 2002 (Figure 3), abundance in 2002 was probably on a par with abundance in 2003 (Figure 7).

Despite the similarity between our results and those of Regehr et al. (2010) and Bromaghin et al. (2015), there are some important differences between both the data and the methods used among the investigations. The prior investigations included fall captures that occurred in some of the earliest years of the study period and amalgamated any spring and fall observations of a bear into a single observation for the year. Telemetry‐assisted captures were also incorporated as mark–recapture observations in the prior investigations. In this study, we used spring data only and excluded telemetry‐assisted captures to eliminate sources of heterogeneity in recapture probabilities. In addition, the prior investigations used covariates, such as various measures of sea ice availability or sampling effort, to explain annual variation in survival and recapture probabilities. We took a simplified approach in this study, using a small number of (noncovariate) models with considerable flexibility for survival and recapture probabilities to vary through time and fit whatever patterns existed in the data. Although our approach precludes us from drawing direct inferences about potential links between covariates and survival, it results in potentially improved fit to the data and does not preclude subsequent comparisons of parameter estimates with other data sources.

The multistate structure of our CJS model seems to be an improvement compared with a traditional CJS model (e.g., Schaub et al., 2004) for the SBS polar bear subpopulation. As discussed above, prior mark–recapture analyses of SBS data used various covariates to explain heterogeneity in recapture probabilities. Our system of five spatial states (Figure 2) was designed to parsimoniously approximate key aspects of bear movement patterns most pertinent to our investigation: a bear's availability for capture, use of the narrow continental shelf on the east versus the broader shelf to the west, and use of nearshore areas with high‐quality habitat that tend to receive more search effort than offshore areas. The multistate model seems preferable to the use of traditional covariates because its structure directly accounts for biological and sampling mechanisms that may underlie at least some of the heterogeneity in recapture probabilities. An additional advantage of the multistate design is that it allowed us to incorporate location information from telemetry‐instrumented bears into mark–recapture models to better inform estimation of state transition.

The design of the states was an important aspect of the model. We selected the division between nearshore and offshore states with the expectation that time‐structured recapture probabilities would be lower in the offshore states, thereby facilitating the incorporation of heterogeneity into the model. Our approach was only partially successful. Estimated recapture probabilities in the Offshore‐west state were sometimes so low that they induced numerical instability in the abundance estimates (from division by small probabilities). This was much less of a problem in our top model, but a hint of such instability can be seen in the long upper tail in the boxplot for abundance in 2003 (Figure 7). Conversely, estimated recapture probabilities were unexpectedly similar in the Nearshore‐east and Offshore‐east states. We therefore incorporated heterogeneity in recapture probabilities into the model, but not quite as we anticipated, and future analyses using spatial structure might benefit from a reconsideration of state definitions.

The polar bear research program in Alaska continues to provide valuable insights into subpopulation dynamics and ecological drivers in a rapidly changing environment, and in the absence of coordinated research in both Alaska and Canada, information from the Alaska SBS should prove useful for management of the entire SBS subpopulation. A majority of the subpopulation range lies offshore of the Alaska coast (west of 141°W), and abundance estimates for the Alaska SBS from 2002 to 2010 averaged 65% of the estimates for the entire subpopulation (Bromaghin et al., 2015). Comanagement authorities moved the boundary between the SBS and NBS subpopulations westward from 125° W to 133° W in 2014, so Alaska habitat now constitutes an even larger percentage of the subpopulation range. Consequently, the abundance of SBS polar bears in Alaska and the entire subpopulation can be expected to trend similarly simply because the Alaska component is such a large share of the whole. In addition, although vital rates might differ between the Alaska and Canada portions of the SBS in some years, existing evidence indicates that the two subpopulation components have responded similar to contemporary ecosystem drivers (Bromaghin et al., 2015).

## 5 CONCLUSIONS

The polar bear carrying capacity of the SBS has been eroding for nearly two decades. Subpopulation abundance increased after the 1972 passage of the MMPA curtailed sport harvest in Alaska (Amstrup et al., 1986), and growth may have continued as late as 1998 (Amstrup et al., 2001). However, subsequent research suggests that the subpopulation has since been in general decline, with large reductions appearing to occur during punctuated periods of low survival. Abundance estimates in the late 1990s (Amstrup et al., 2001) and 2004 (Bromaghin et al., 2015) imply that subpopulation size likely fell during that interval, though estimates of high survival from 2001 to 2003 (Bromaghin et al., 2015; Regehr et al., 2010; this study) suggest that any decline must have occurred prior to 2001. Regehr et al. (2010) found that a second period of low survival began in 2004. Bromaghin et al. (2015) confirmed that low survival began in 2004 and largely persisted through 2009. Our current results for the Alaska SBS corroborate low survival from 2004 to 2009 and additionally suggest that survival was again poor in 2012. A pattern of relative stability interspersed with periods of low survival and population decline is a plausible trajectory for a specialized apex predator whose niche habitat is vanishing. Our finding that abundance was relatively stable from 2006 to 2015 (2012 excepted) is encouraging, but the general level of abundance over this period is probably lower than at any time since passage of the MMPA. Given climate model projections for continued global warming and sea ice loss (e.g., SIMIP Community, 2020), further reductions in the abundance of polar bears in the SBS can be expected in the future (e.g., Atwood, Marcot, et al., 2016; Molnár et al., 2020).

## CONFLICT OF INTEREST

None declared.

## AUTHOR CONTRIBUTIONS

**Jeffrey F. Bromaghin:** Conceptualization (lead); Data curation (equal); Formal analysis (lead); Methodology (lead); Project administration (lead); Writing‐original draft (lead); Writing‐review & editing (lead). **David C. Douglas:** Conceptualization (supporting); Data curation (supporting); Methodology (supporting); Writing‐review & editing (supporting). **George M. Durner:** Conceptualization (supporting); Data curation (supporting); Investigation (supporting); Writing‐review & editing (supporting). **Kristin S. Simac:** Data curation (supporting); Investigation (supporting); Writing‐review & editing (supporting). **Todd C. Atwood:** Conceptualization (supporting); Data curation (supporting); Formal analysis (supporting); Investigation (supporting); Writing‐review & editing (supporting).

## Supporting information

Appendix S1

## Data Availability

The data are archived in Bromaghin et al. (2020) at: https://doi.org/10.5066/P9A9E5UP.
