# Supplementary material for: Survival and abundance of polar bears in Alaska’s Beaufort Sea, 2001–2016
Source: Ecol Evol. 2021 Sep 23;11(20):14250–67. doi: 10.1002/ece3.8139 (PMC8525099; doi:10.1002/ece3.8139)
Supplement: Supplementary file 1 — Appendix S1 [file ECE3-11-14250-s001.docx]

Appendix

The appendix contains details and supplementary information that are not necessary to understand the primary findings and conclusions of our investigation, but that nevertheless might be of interest to some readers or of value to future investigations.

Catch statistics

The total number of polar bears of each sex and age category captured in each year, as well as the number of recaptures of previously observed individuals, are tabulated in Table A1. The sums of all captures and recaptures across all age classes includes individuals whose ages were not known, the majority of which were never physically captured but rather identified from genetic samples obtained via biopsy darting. Similarly, the sums across sex classes include one individual whose sex was unknown. The number of times that individual bears were captured is summarized in Table A2. The number of observations in each state are presented in Table A3, along with the state in which each individual was observed (or not observed) the following year.

Statistics summarizing posterior densities

We drew samples of 20,000 values from the posterior distributions of the primary parameters of interest and derived quantities. Such large samples were taken to support additional research planned in the future. For each sample, we computed the mean (Mean), standard deviation (SD), and the 2.5% (Q_0.025_), 25% (Q_0.25_), 50% (Q_0.5_), 75% (Q_0.75_), and 97.5% (Q_0.975_) quantiles. Statistics for the posterior distributions of survival probabilities for each age class, by sex and year, are in tables A4-A10; statistics for the spatial state transition probabilities are in Table A11; statistics for the proportion of marked bears estimated to be in spatial states 1-4 each year are in Table A12; statistics for the recapture probabilities by state and year are in Table A13; and statistics for annual abundance estimates are in Table A14.

Population projections

We compared the temporal trend in abundance estimates with the abundance trajectory of a population projection as an informal consistency check between estimates of abundance and survival probabilities. Unmodeled heterogeneity in either recapture or survival probabilities can bias parameter estimates (e.g., Carothers 1979; Abadi et al. 2013) and the Horvitz-Thompson abundance estimator is derived directly from recapture probabilities (McDonald and Amstrup 2001), so any bias in estimates of recapture probability propagate to abundance estimates. Consequently, unmodeled heterogeneity could cause the temporal trend in abundance estimates (based primarily on recapture probabilities) to differ from the trend in projected abundance (based largely on survival probabilities). Our methods closely follow those of Bromaghin et al. (2015, Appendix E) and are only briefly summarized here.

The 2001-2016 mark-recapture data were used to estimate the distributions of age-0 cub litter size (DLS) and of bear groups (DBG) with respect to sex, age, and family status (age and number of dependent young). For the DLS, the number of age-0 litters of size 1, 2, and 3 were counted and the counts were converted to proportions to form the DLS. For the DBG, females and males ≥ age 2 were classified by age; the maximum observed age of 29 was used as the maximum for both sexes. Females ≥ age 5 were additionally classified by the number and age of their dependent young (no dependent young, 1-3 age-0 cubs or 1-3 age-1 yearlings). For the purposes of this exercise, age-2 young accompanying an adult female were treated as independent observations and tallied separately. The observations in each of these 206 group categories were counted and the count of each category was increased by 1 so that all categories would have a non-zero probability of occurring. The adjusted counts were converted to proportions to form the DBG.

We used a simple reproductive model based on the DLS and annual breeding probabilities derived from the mark-recapture data. For each year 2002 to 2015, a breeding probability $B_{y}$ was computed as

$$B_{k}=\frac{Number of females with age-0 cubs in year k}{Number of females with age-0 cubs and lone females in year k}$$

The resulting breeding probabilities are presented in Figure A1.

The composition of the population was randomly initialized in year 1 (2001) using the

DBG. We arbitrarily decided to start the projection with approximately 1,000 bears in the population and the expected group size based on the DBG was 1.523, so a fixed sample size of 657 groups had an expected population size of 1000. We initialized the population in year 1 by drawing a multinomial sample of 657 groups from the DBG. Note that the trajectory (relative change) in abundance through time is the key information, so the starting population size is immaterial as long as it is large enough to incorporate the range of diversity in the DBG.

The initial population of bears in year 1 was projected forward one year at a time for 14 years (to 2015). The existing population of bears in any year k was projected forward to year k+1 using a sample of survival probabilities drawn from the posterior distributions for year k. The survival of each individual bear was determined with an independent random draw from a Bernoulli density. If a female with dependent young in year k did not survive to year k+1, neither did her young. Females of age 4 or older in year k available to reproduce (without dependent young or with age-2 young) that survived to year k+1 acquired a new litter of age-0 cubs in year k+1 with probability $B_{k+1}$. The number of age-0 cubs in a new litter was established by a draw from the DLS. An individual that survived to the maximum age of 29 in year k did not survive to year k+1. All age-1 yearlings in year k that survived to age-2 in year k+1 were randomly assigned a sex with an independent draw from a Bernoulli density with mean 0.5.

The population projection was replicated 20,000 times, once for each sample from the

survival probability posterior distributions. Because of the sex, age class, and temporal structure of the survival probabilities (see Materials and methods), each sample from the survival probability posterior distributions consisted of a suite of linked survival probabilities that maintained the sex, age class, and temporal structure. In order to most easily compare the trends in estimated and projected abundance, we computed the mean abundance in each year across the 20,000 projections and then computed the multiplicative scaling factor that minimized the squared differences between the mean projected abundance and the multistate CJS abundance estimates. The resulting scaling factor was then applied to the abundance of all projections. We summarized the scaled projections by computing the mean and the range of the central 50% and 95% of the values (Figure A2). The correlation between our abundance estimates and the mean projected abundance was 0.845.

The similarity of the trends in abundance estimates and projected abundance overall does

not raise any substantial concerns regarding the abundance or survival probability estimates. Projected abundance is substantially greater than estimated abundance in 2002, which is consistent with our conclusion that the 2002 estimated was negatively biased by known factors and the first admissible abundance estimate is in 2003 (see Discussion). The decline in abundance from 2003 to 2009 is similar in the two series, though the decline in projected abundance appears delayed. Estimated abundance increased from 2009 to 2012, which seems consistent with the estimated survival probabilities, but projected abundance appears essentially stable for reasons that aren’t clear. Both series decline by approximately equal amounts from 2012 to 2013, consistent with low survival in 2012, and then remain approximately stable through 2015.

Literature cited

Abadi, F., Botha, A. & Altwegg, R. (2013) Revisiting the effect of capture heterogeneity on survival estimates in capture-mark-recapture studies: Does it matter? *PLoS ONE*, 8, e62636.

Bromaghin, J.F., McDonald, T.L., Stirling, I., Derocher, A.E., Richardson, E.S., Regehr, E.V., Douglas, D.C., Durner, G.M., Atwood, T. & Amstrup, S.C. (2015) Polar bear population dynamics in the southern Beaufort Sea during a period of sea ice decline*. Ecological Applications*, 25, 634-651.

Carothers, A.D. (1979) Quantifying unequal catchability and its effect on survival estimates in an actual population. *Journal of Animal Ecology*, 48, 863–869.

McDonald, T.L. & Amstrup, S.C. (2001) Estimation of population size using open capture–recapture models*. Journal of Agricultural, Biological, and Environmental Statistics*, 6, 206-220.

| Table S1. The number of southern Beaufort Sea polar bear mark-recapture observations in Alaska from 2001 to 2016 (C) and the number of those individuals that were recaptures (R), by age class, sex, and year. The totals include individuals whose sex or age were unknown. | | | | | | | | | | | | | | | |
| --- | --- | --- | --- | --- | --- | --- | --- | --- | --- | --- | --- | --- | --- | --- | --- |
|  |  | Age 0 | | Age 1 | | Age 2 | | Age 3-4 | | Age 5-19 | | Age 20+ | | Total | |
| Sex | Year | C | R | C | R | C | R | C | R | C | R | C | R | C | R |
| Female | 2001 | 6 | 0 | 8 | 0 | 0 | 0 | 3 | 0 | 19 | 0 | 2 | 0 | 39 | 0 |
| Female | 2002 | 7 | 0 | 2 | 0 | 4 | 1 | 5 | 1 | 23 | 4 | 3 | 0 | 44 | 6 |
| Female | 2003 | 2 | 0 | 1 | 0 | 2 | 0 | 5 | 0 | 20 | 2 | 1 | 0 | 33 | 2 |
| Female | 2004 | 10 | 0 | 1 | 0 | 4 | 0 | 12 | 1 | 32 | 7 | 4 | 2 | 65 | 10 |
| Female | 2005 | 7 | 0 | 1 | 0 | 2 | 0 | 5 | 0 | 18 | 5 | 4 | 1 | 38 | 6 |
| Female | 2006 | 4 | 0 | 6 | 1 | 6 | 0 | 3 | 0 | 21 | 10 | 5 | 1 | 47 | 12 |
| Female | 2007 | 5 | 0 | 4 | 0 | 1 | 0 | 1 | 0 | 20 | 5 | 2 | 2 | 34 | 8 |
| Female | 2008 | 5 | 0 | 0 | 0 | 1 | 0 | 9 | 1 | 22 | 8 | 3 | 1 | 40 | 10 |
| Female | 2009 | 7 | 0 | 5 | 1 | 0 | 0 | 1 | 0 | 27 | 14 | 1 | 1 | 41 | 16 |
| Female | 2010 | 5 | 0 | 2 | 0 | 2 | 1 | 0 | 0 | 22 | 11 | 0 | 0 | 31 | 12 |
| Female | 2011 | 4 | 0 | 5 | 1 | 2 | 1 | 8 | 3 | 24 | 14 | 4 | 2 | 55 | 21 |
| Female | 2012 | 0 | 0 | 8 | 1 | 1 | 0 | 7 | 0 | 19 | 12 | 2 | 1 | 45 | 15 |
| Female | 2013 | 3 | 0 | 0 | 0 | 3 | 0 | 3 | 0 | 24 | 16 | 0 | 0 | 42 | 16 |
| Female | 2014 | 3 | 0 | 2 | 0 | 3 | 0 | 5 | 3 | 13 | 7 | 0 | 0 | 28 | 10 |
| Female | 2015 | 0 | 0 | 2 | 0 | 3 | 1 | 6 | 1 | 10 | 5 | 0 | 0 | 22 | 7 |
| Female | 2016 | 0 | 0 | 0 | 0 | 1 | 0 | 0 | 0 | 7 | 2 | 1 | 0 | 9 | 2 |
| Male | 2001 | 5 | 0 | 2 | 0 | 1 | 0 | 2 | 0 | 13 | 0 | 1 | 0 | 24 | 0 |
| Male | 2002 | 3 | 0 | 4 | 1 | 6 | 0 | 3 | 0 | 17 | 0 | 1 | 1 | 34 | 2 |
| Male | 2003 | 8 | 0 | 5 | 0 | 1 | 0 | 6 | 3 | 34 | 3 | 4 | 1 | 59 | 7 |
| Male | 2004 | 10 | 0 | 5 | 1 | 6 | 0 | 7 | 4 | 30 | 7 | 1 | 0 | 59 | 12 |
| Male | 2005 | 14 | 0 | 0 | 0 | 2 | 2 | 3 | 1 | 20 | 11 | 3 | 2 | 42 | 16 |
| Male | 2006 | 4 | 0 | 0 | 0 | 0 | 0 | 4 | 0 | 30 | 19 | 0 | 0 | 38 | 19 |
| Male | 2007 | 7 | 0 | 2 | 0 | 2 | 0 | 1 | 0 | 28 | 13 | 1 | 1 | 42 | 14 |
| Male | 2008 | 6 | 0 | 1 | 0 | 0 | 0 | 4 | 0 | 24 | 12 | 3 | 3 | 38 | 15 |
| Male | 2009 | 9 | 0 | 6 | 0 | 0 | 0 | 0 | 0 | 33 | 19 | 2 | 1 | 51 | 20 |
| Male | 2010 | 8 | 0 | 5 | 0 | 2 | 2 | 1 | 0 | 18 | 15 | 0 | 0 | 34 | 17 |
| Male | 2011 | 1 | 0 | 5 | 1 | 6 | 3 | 5 | 1 | 20 | 11 | 2 | 1 | 45 | 17 |
| Male | 2012 | 4 | 0 | 2 | 1 | 4 | 2 | 6 | 2 | 27 | 18 | 3 | 3 | 53 | 27 |
| Male | 2013 | 4 | 0 | 2 | 0 | 3 | 0 | 8 | 1 | 17 | 13 | 3 | 3 | 43 | 17 |
| Male | 2014 | 3 | 0 | 3 | 0 | 0 | 0 | 3 | 2 | 10 | 7 | 1 | 1 | 20 | 10 |
| Male | 2015 | 3 | 0 | 2 | 0 | 0 | 0 | 6 | 0 | 5 | 5 | 0 | 0 | 16 | 5 |
| Male | 2016 | 0 | 0 | 2 | 0 | 1 | 0 | 2 | 1 | 6 | 3 | 1 | 1 | 12 | 5 |
| All | 2001 | 11 | 0 | 10 | 0 | 1 | 0 | 5 | 0 | 32 | 0 | 3 | 0 | 63 | 0 |
| All | 2002 | 10 | 0 | 6 | 1 | 10 | 1 | 8 | 1 | 40 | 4 | 4 | 1 | 78 | 8 |
| All | 2003 | 10 | 0 | 6 | 0 | 3 | 0 | 11 | 3 | 54 | 5 | 5 | 1 | 92 | 9 |
| All | 2004 | 20 | 0 | 6 | 1 | 10 | 0 | 19 | 5 | 62 | 14 | 5 | 2 | 124 | 22 |
| All | 2005 | 21 | 0 | 1 | 0 | 4 | 2 | 8 | 1 | 38 | 16 | 7 | 3 | 80 | 22 |
| All | 2006 | 8 | 0 | 6 | 1 | 6 | 0 | 7 | 0 | 51 | 29 | 5 | 1 | 85 | 31 |
| All | 2007 | 12 | 0 | 6 | 0 | 3 | 0 | 2 | 0 | 48 | 18 | 3 | 3 | 76 | 22 |
| All | 2008 | 11 | 0 | 1 | 0 | 1 | 0 | 13 | 1 | 46 | 20 | 6 | 4 | 78 | 25 |
| All | 2009 | 16 | 0 | 11 | 1 | 0 | 0 | 1 | 0 | 60 | 33 | 3 | 2 | 92 | 36 |
| All | 2010 | 13 | 0 | 7 | 0 | 4 | 3 | 1 | 0 | 40 | 26 | 0 | 0 | 65 | 29 |
| All | 2011 | 5 | 0 | 10 | 2 | 8 | 4 | 13 | 4 | 44 | 25 | 6 | 3 | 100 | 38 |
| All | 2012 | 4 | 0 | 10 | 2 | 5 | 2 | 13 | 2 | 46 | 30 | 5 | 4 | 98 | 42 |
| All | 2013 | 7 | 0 | 2 | 0 | 6 | 0 | 11 | 1 | 41 | 29 | 3 | 3 | 86 | 33 |
| All | 2014 | 6 | 0 | 5 | 0 | 3 | 0 | 8 | 5 | 23 | 14 | 1 | 1 | 48 | 20 |
| All | 2015 | 3 | 0 | 4 | 0 | 3 | 1 | 12 | 1 | 15 | 10 | 0 | 0 | 38 | 12 |
| All | 2016 | 0 | 0 | 2 | 0 | 2 | 0 | 2 | 1 | 13 | 5 | 2 | 1 | 21 | 7 |

| Table S2. The number of times individual bears were observed during the mark-recapture investigation in the Alaska portion of the southern Beaufort Sea from 2001 to 2016. | | |
| --- | --- | --- |
| Times  captured |  | Number  of bears |
| 1 |  | 657 |
| 2 |  | 131 |
| 3 |  | 44 |
| 4 |  | 17 |
| 5 |  | 12 |
| 6 |  | 5 |
| 7 |  | 1 |
| 8 |  | 1 |

| Table S3. The number of southern Beaufort Sea polar bears released in each state from 2001 to 2015 (N) and the state in which they were observed the following year for the mark-recapture (MR) and telemetry (Tel) data sets. States were State 1: Nearshore-west, State 2: Nearshore-east, State 3: Offshore-west, State 4: Offshore-east, and State 5: Elsewhere, with the state of bears that were not observed the following year being unknown (U). | | | | | | | | |
| --- | --- | --- | --- | --- | --- | --- | --- | --- |
|  |  |  | Observed state the following year | | | | | |
| Data | State | N | 1 | 2 | 3 | 4 | 5 | U |
| MR | 1 | 335 | 18 | 7 | 0 | 5 | 0 | 305 |
|  | 2 | 524 | 3 | 42 | 2 | 15 | 0 | 462 |
|  | 3 | 63 | 6 | 0 | 0 | 0 | 0 | 57 |
|  | 4 | 281 | 3 | 13 | 0 | 12 | 0 | 253 |
|  | 5 | 0 | 0 | 0 | 0 | 0 | 0 | 0 |
|  |  |  |  |  |  |  |  |  |
| Tel | 1 | 26 | 4 | 0 | 1 | 1 | 8 | 12 |
|  | 2 | 80 | 0 | 24 | 3 | 7 | 9 | 37 |
|  | 3 | 20 | 2 | 1 | 0 | 3 | 5 | 9 |
|  | 4 | 64 | 2 | 7 | 2 | 3 | 28 | 22 |
|  | 5 | 145 | 4 | 4 | 5 | 7 | 56 | 69 |

| Table S4. Statistics summarizing samples of 20,000 from the posterior distributions of survival probabilities for polar bears in the Alaska portion of the southern Beaufort Sea in the Age0 age class by sex and year, including the mean (Mean), standard deviation (SD), and the 2.5% (Q_0.025_), 25% (Q_0.25_), 50% (Q_0.5_), 75% (Q_0.75_), and 97.5% (Q_0.975_) quantiles. Survival could not be estimated in 2015 because of the usual confounding between recapture and survival probabilities in time-structured mark-recapture models. | | | | | | | | |
| --- | --- | --- | --- | --- | --- | --- | --- | --- |
| Sex | Year | Mean | SD | Q_0.025_ | Q_0.25_ | Q_0.5_ | Q_0.75_ | Q_0.975_ |
| Female | 2001 | 0.6177 | 0.1760 | 0.2521 | 0.4916 | 0.6382 | 0.7596 | 0.8942 |
| Female | 2002 | 0.6394 | 0.1722 | 0.2729 | 0.5200 | 0.6644 | 0.7780 | 0.8980 |
| Female | 2003 | 0.5529 | 0.1989 | 0.1789 | 0.3966 | 0.5606 | 0.7146 | 0.8855 |
| Female | 2004 | 0.3244 | 0.2071 | 0.0637 | 0.1629 | 0.2700 | 0.4427 | 0.8259 |
| Female | 2005 | 0.1745 | 0.1213 | 0.0416 | 0.0930 | 0.1420 | 0.2172 | 0.5091 |
| Female | 2006 | 0.3721 | 0.2242 | 0.0641 | 0.1878 | 0.3256 | 0.5274 | 0.8511 |
| Female | 2007 | 0.2997 | 0.1950 | 0.0589 | 0.1508 | 0.2479 | 0.3994 | 0.7936 |
| Female | 2008 | 0.2497 | 0.1590 | 0.0569 | 0.1342 | 0.2089 | 0.3215 | 0.6776 |
| Female | 2009 | 0.6256 | 0.1671 | 0.2755 | 0.5066 | 0.6413 | 0.7592 | 0.8918 |
| Female | 2010 | 0.6468 | 0.1650 | 0.2896 | 0.5344 | 0.6700 | 0.7770 | 0.8955 |
| Female | 2011 | 0.6089 | 0.1943 | 0.1953 | 0.4725 | 0.6424 | 0.7653 | 0.8933 |
| Female | 2012 | 0.0912 | 0.0895 | 0.0175 | 0.0405 | 0.0655 | 0.1076 | 0.3323 |
| Female | 2013 | 0.4703 | 0.2451 | 0.0722 | 0.2552 | 0.4643 | 0.6833 | 0.8841 |
| Female | 2014 | 0.4838 | 0.2575 | 0.0491 | 0.2574 | 0.4969 | 0.7126 | 0.8879 |
| Male | 2001 | 0.6177 | 0.1760 | 0.2521 | 0.4916 | 0.6382 | 0.7596 | 0.8942 |
| Male | 2002 | 0.6394 | 0.1722 | 0.2729 | 0.5200 | 0.6644 | 0.7780 | 0.8980 |
| Male | 2003 | 0.5529 | 0.1989 | 0.1789 | 0.3966 | 0.5606 | 0.7146 | 0.8855 |
| Male | 2004 | 0.3244 | 0.2071 | 0.0637 | 0.1629 | 0.2700 | 0.4427 | 0.8259 |
| Male | 2005 | 0.1745 | 0.1213 | 0.0416 | 0.0930 | 0.1420 | 0.2172 | 0.5091 |
| Male | 2006 | 0.3721 | 0.2242 | 0.0641 | 0.1878 | 0.3256 | 0.5274 | 0.8511 |
| Male | 2007 | 0.2997 | 0.1950 | 0.0589 | 0.1508 | 0.2479 | 0.3994 | 0.7936 |
| Male | 2008 | 0.2497 | 0.1590 | 0.0569 | 0.1342 | 0.2089 | 0.3215 | 0.6776 |
| Male | 2009 | 0.6256 | 0.1671 | 0.2755 | 0.5066 | 0.6413 | 0.7592 | 0.8918 |
| Male | 2010 | 0.6468 | 0.1650 | 0.2896 | 0.5344 | 0.6700 | 0.7770 | 0.8955 |
| Male | 2011 | 0.6089 | 0.1943 | 0.1953 | 0.4725 | 0.6424 | 0.7653 | 0.8933 |
| Male | 2012 | 0.0912 | 0.0895 | 0.0175 | 0.0405 | 0.0655 | 0.1076 | 0.3323 |
| Male | 2013 | 0.4703 | 0.2451 | 0.0722 | 0.2552 | 0.4643 | 0.6833 | 0.8841 |
| Male | 2014 | 0.4838 | 0.2575 | 0.0491 | 0.2574 | 0.4969 | 0.7126 | 0.8879 |

| Table S5. Statistics summarizing samples of 20,000 from the posterior distributions of survival probabilities for polar bears in the Alaska portion of the southern Beaufort Sea in the Age1 age class by sex and year, including the mean (Mean), standard deviation (SD), and the 2.5% (Q_0.025_), 25% (Q_0.25_), 50% (Q_0.5_), 75% (Q_0.75_), and 97.5% (Q_0.975_) quantiles. Survival could not be estimated in 2015 because of the usual confounding between recapture and survival probabilities in time-structured mark-recapture models. | | | | | | | | |
| --- | --- | --- | --- | --- | --- | --- | --- | --- |
| Sex | Year | Mean | SD | Q_0.025_ | Q_0.25_ | Q_0.5_ | Q_0.75_ | Q_0.975_ |
| Female | 2001 | 0.8482 | 0.1158 | 0.5483 | 0.7919 | 0.8827 | 0.9354 | 0.9779 |
| Female | 2002 | 0.8601 | 0.1105 | 0.5613 | 0.8109 | 0.8943 | 0.9415 | 0.9796 |
| Female | 2003 | 0.8092 | 0.1367 | 0.4711 | 0.7337 | 0.8450 | 0.9170 | 0.9732 |
| Female | 2004 | 0.6161 | 0.1829 | 0.2614 | 0.4811 | 0.6209 | 0.7597 | 0.9305 |
| Female | 2005 | 0.4341 | 0.2114 | 0.1216 | 0.2628 | 0.4004 | 0.5823 | 0.8892 |
| Female | 2006 | 0.6551 | 0.1967 | 0.2547 | 0.5107 | 0.6717 | 0.8188 | 0.9553 |
| Female | 2007 | 0.5807 | 0.2191 | 0.1905 | 0.4040 | 0.5805 | 0.7635 | 0.9504 |
| Female | 2008 | 0.5348 | 0.1971 | 0.1951 | 0.3811 | 0.5261 | 0.6807 | 0.9162 |
| Female | 2009 | 0.8562 | 0.1036 | 0.5921 | 0.8060 | 0.8846 | 0.9339 | 0.9770 |
| Female | 2010 | 0.8668 | 0.0996 | 0.6033 | 0.8208 | 0.8954 | 0.9401 | 0.9782 |
| Female | 2011 | 0.8379 | 0.1355 | 0.4701 | 0.7809 | 0.8838 | 0.9371 | 0.9782 |
| Female | 2012 | 0.2680 | 0.1592 | 0.0687 | 0.1516 | 0.2304 | 0.3466 | 0.6876 |
| Female | 2013 | 0.7256 | 0.2170 | 0.2156 | 0.5890 | 0.7898 | 0.9056 | 0.9737 |
| Female | 2014 | 0.7341 | 0.2129 | 0.2173 | 0.6084 | 0.8028 | 0.9063 | 0.9715 |
| Male | 2001 | 0.8482 | 0.1158 | 0.5483 | 0.7919 | 0.8827 | 0.9354 | 0.9779 |
| Male | 2002 | 0.8601 | 0.1105 | 0.5613 | 0.8109 | 0.8943 | 0.9415 | 0.9796 |
| Male | 2003 | 0.8092 | 0.1367 | 0.4711 | 0.7337 | 0.8450 | 0.9170 | 0.9732 |
| Male | 2004 | 0.6161 | 0.1829 | 0.2614 | 0.4811 | 0.6209 | 0.7597 | 0.9305 |
| Male | 2005 | 0.4341 | 0.2114 | 0.1216 | 0.2628 | 0.4004 | 0.5823 | 0.8892 |
| Male | 2006 | 0.6551 | 0.1967 | 0.2547 | 0.5107 | 0.6717 | 0.8188 | 0.9553 |
| Male | 2007 | 0.5807 | 0.2191 | 0.1905 | 0.4040 | 0.5805 | 0.7635 | 0.9504 |
| Male | 2008 | 0.5348 | 0.1971 | 0.1951 | 0.3811 | 0.5261 | 0.6807 | 0.9162 |
| Male | 2009 | 0.8562 | 0.1036 | 0.5921 | 0.8060 | 0.8846 | 0.9339 | 0.9770 |
| Male | 2010 | 0.8668 | 0.0996 | 0.6033 | 0.8208 | 0.8954 | 0.9401 | 0.9782 |
| Male | 2011 | 0.8379 | 0.1355 | 0.4701 | 0.7809 | 0.8838 | 0.9371 | 0.9782 |
| Male | 2012 | 0.2680 | 0.1592 | 0.0687 | 0.1516 | 0.2304 | 0.3466 | 0.6876 |
| Male | 2013 | 0.7256 | 0.2170 | 0.2156 | 0.5890 | 0.7898 | 0.9056 | 0.9737 |
| Male | 2014 | 0.7341 | 0.2129 | 0.2173 | 0.6084 | 0.8028 | 0.9063 | 0.9715 |

| Table S6. Statistics summarizing samples of 20,000 from the posterior distributions of survival probabilities for polar bears in the Alaska portion of the southern Beaufort Sea in the Age2 age class by sex and year, including the mean (Mean), standard deviation (SD), and the 2.5% (Q_0.025_), 25% (Q_0.25_), 50% (Q_0.5_), 75% (Q_0.75_), and 97.5% (Q_0.975_) quantiles. Survival could not be estimated in 2015 because of the usual confounding between recapture and survival probabilities in time-structured mark-recapture models. | | | | | | | | |
| --- | --- | --- | --- | --- | --- | --- | --- | --- |
| Sex | Year | Mean | SD | Q_0.025_ | Q_0.25_ | Q_0.5_ | Q_0.75_ | Q_0.975_ |
| Female | 2001 | 0.8730 | 0.1008 | 0.6038 | 0.8327 | 0.9048 | 0.9453 | 0.9769 |
| Female | 2002 | 0.8868 | 0.0845 | 0.6610 | 0.8499 | 0.9123 | 0.9482 | 0.9776 |
| Female | 2003 | 0.8370 | 0.1203 | 0.5299 | 0.7769 | 0.8699 | 0.9301 | 0.9748 |
| Female | 2004 | 0.6483 | 0.1919 | 0.2696 | 0.5053 | 0.6587 | 0.8042 | 0.9586 |
| Female | 2005 | 0.4750 | 0.1757 | 0.1897 | 0.3455 | 0.4528 | 0.5851 | 0.8679 |
| Female | 2006 | 0.6912 | 0.1863 | 0.2982 | 0.5594 | 0.7104 | 0.8480 | 0.9625 |
| Female | 2007 | 0.6284 | 0.1784 | 0.2841 | 0.4974 | 0.6295 | 0.7651 | 0.9465 |
| Female | 2008 | 0.5743 | 0.1970 | 0.2184 | 0.4244 | 0.5690 | 0.7249 | 0.9397 |
| Female | 2009 | 0.8772 | 0.0968 | 0.6152 | 0.8369 | 0.9074 | 0.9463 | 0.9775 |
| Female | 2010 | 0.8877 | 0.0891 | 0.6444 | 0.8524 | 0.9159 | 0.9509 | 0.9786 |
| Female | 2011 | 0.8602 | 0.1249 | 0.5151 | 0.8130 | 0.9053 | 0.9494 | 0.9788 |
| Female | 2012 | 0.2988 | 0.1495 | 0.0954 | 0.1923 | 0.2691 | 0.3721 | 0.6827 |
| Female | 2013 | 0.7660 | 0.1785 | 0.3339 | 0.6568 | 0.8160 | 0.9125 | 0.9711 |
| Female | 2014 | 0.7579 | 0.2187 | 0.2011 | 0.6430 | 0.8390 | 0.9289 | 0.9752 |
| Male | 2001 | 0.9094 | 0.0784 | 0.6934 | 0.8833 | 0.9349 | 0.9631 | 0.9844 |
| Male | 2002 | 0.9202 | 0.0640 | 0.7474 | 0.8965 | 0.9402 | 0.9652 | 0.9844 |
| Male | 2003 | 0.8817 | 0.0954 | 0.6314 | 0.8390 | 0.9109 | 0.9525 | 0.9829 |
| Male | 2004 | 0.7237 | 0.1678 | 0.3629 | 0.6073 | 0.7444 | 0.8609 | 0.9717 |
| Male | 2005 | 0.5647 | 0.1709 | 0.2564 | 0.4417 | 0.5549 | 0.6822 | 0.9110 |
| Male | 2006 | 0.7597 | 0.1627 | 0.3849 | 0.6546 | 0.7877 | 0.8953 | 0.9750 |
| Male | 2007 | 0.7073 | 0.1587 | 0.3735 | 0.5970 | 0.7194 | 0.8312 | 0.9634 |
| Male | 2008 | 0.6563 | 0.1822 | 0.2929 | 0.5250 | 0.6664 | 0.8007 | 0.9590 |
| Male | 2009 | 0.9128 | 0.0744 | 0.7053 | 0.8868 | 0.9370 | 0.9636 | 0.9846 |
| Male | 2010 | 0.9205 | 0.0684 | 0.7288 | 0.8971 | 0.9429 | 0.9669 | 0.9853 |
| Male | 2011 | 0.8992 | 0.0977 | 0.6220 | 0.8691 | 0.9357 | 0.9655 | 0.9853 |
| Male | 2012 | 0.3809 | 0.1621 | 0.1352 | 0.2628 | 0.3564 | 0.4737 | 0.7694 |
| Male | 2013 | 0.8216 | 0.1518 | 0.4260 | 0.7430 | 0.8702 | 0.9409 | 0.9799 |
| Male | 2014 | 0.8097 | 0.1948 | 0.2696 | 0.7332 | 0.8880 | 0.9521 | 0.9831 |

| Table S7. Statistics summarizing samples of 20,000 from the posterior distributions of survival probabilities for polar bears in the Alaska portion of the southern Beaufort Sea in the Ages3-4 age class by sex and year, including the mean (Mean), standard deviation (SD), and the 2.5% (Q_0.025_), 25% (Q_0.25_), 50% (Q_0.5_), 75% (Q_0.75_), and 97.5% (Q_0.975_) quantiles. Survival could not be estimated in 2015 because of the usual confounding between recapture and survival probabilities in time-structured mark-recapture models. | | | | | | | | |
| --- | --- | --- | --- | --- | --- | --- | --- | --- |
| Sex | Year | Mean | SD | Q_0.025_ | Q_0.25_ | Q_0.5_ | Q_0.75_ | Q_0.975_ |
| Female | 2001 | 0.9295 | 0.0583 | 0.7712 | 0.9084 | 0.9485 | 0.9698 | 0.9856 |
| Female | 2002 | 0.9378 | 0.0478 | 0.8081 | 0.9189 | 0.9530 | 0.9716 | 0.9858 |
| Female | 2003 | 0.9076 | 0.0693 | 0.7311 | 0.8730 | 0.9272 | 0.9606 | 0.9842 |
| Female | 2004 | 0.7735 | 0.1255 | 0.5162 | 0.6837 | 0.7817 | 0.8749 | 0.9729 |
| Female | 2005 | 0.6162 | 0.1575 | 0.3380 | 0.4990 | 0.6056 | 0.7277 | 0.9326 |
| Female | 2006 | 0.8013 | 0.1313 | 0.5072 | 0.7140 | 0.8230 | 0.9103 | 0.9782 |
| Female | 2007 | 0.7499 | 0.1397 | 0.4667 | 0.6506 | 0.7582 | 0.8628 | 0.9715 |
| Female | 2008 | 0.7093 | 0.1427 | 0.4356 | 0.6042 | 0.7115 | 0.8190 | 0.9611 |
| Female | 2009 | 0.9329 | 0.0530 | 0.7894 | 0.9127 | 0.9498 | 0.9699 | 0.9855 |
| Female | 2010 | 0.9387 | 0.0499 | 0.8013 | 0.9216 | 0.9553 | 0.9728 | 0.9862 |
| Female | 2011 | 0.9199 | 0.0793 | 0.6870 | 0.8979 | 0.9503 | 0.9720 | 0.9864 |
| Female | 2012 | 0.4309 | 0.1486 | 0.2086 | 0.3232 | 0.4063 | 0.5130 | 0.7971 |
| Female | 2013 | 0.8508 | 0.1332 | 0.5025 | 0.7853 | 0.8962 | 0.9534 | 0.9829 |
| Female | 2014 | 0.8420 | 0.1665 | 0.3731 | 0.7832 | 0.9097 | 0.9608 | 0.9845 |
| Male | 2001 | 0.9512 | 0.0431 | 0.8342 | 0.9374 | 0.9655 | 0.9799 | 0.9900 |
| Male | 2002 | 0.9573 | 0.0347 | 0.8629 | 0.9451 | 0.9686 | 0.9812 | 0.9903 |
| Male | 2003 | 0.9353 | 0.0515 | 0.8001 | 0.9118 | 0.9508 | 0.9741 | 0.9891 |
| Male | 2004 | 0.8333 | 0.0990 | 0.6172 | 0.7671 | 0.8436 | 0.9123 | 0.9813 |
| Male | 2005 | 0.6985 | 0.1403 | 0.4264 | 0.5982 | 0.6969 | 0.8026 | 0.9575 |
| Male | 2006 | 0.8531 | 0.1062 | 0.6008 | 0.7889 | 0.8758 | 0.9398 | 0.9854 |
| Male | 2007 | 0.8126 | 0.1150 | 0.5596 | 0.7369 | 0.8256 | 0.9042 | 0.9811 |
| Male | 2008 | 0.7793 | 0.1211 | 0.5264 | 0.6952 | 0.7896 | 0.8746 | 0.9745 |
| Male | 2009 | 0.9538 | 0.0384 | 0.8492 | 0.9405 | 0.9661 | 0.9800 | 0.9901 |
| Male | 2010 | 0.9577 | 0.0367 | 0.8554 | 0.9466 | 0.9701 | 0.9819 | 0.9905 |
| Male | 2011 | 0.9442 | 0.0587 | 0.7728 | 0.9303 | 0.9663 | 0.9812 | 0.9905 |
| Male | 2012 | 0.5239 | 0.1486 | 0.2794 | 0.4160 | 0.5084 | 0.6158 | 0.8602 |
| Male | 2013 | 0.8905 | 0.1070 | 0.5976 | 0.8476 | 0.9293 | 0.9690 | 0.9886 |
| Male | 2014 | 0.8808 | 0.1400 | 0.4685 | 0.8450 | 0.9386 | 0.9739 | 0.9894 |

| Table S8. Statistics summarizing samples of 20,000 from the posterior distributions of survival probabilities for polar bears in the Alaska portion of the southern Beaufort Sea in the Ages5-19 age class by sex and year, including the mean (Mean), standard deviation (SD), and the 2.5% (Q_0.025_), 25% (Q_0.25_), 50% (Q_0.5_), 75% (Q_0.75_), and 97.5% (Q_0.975_) quantiles. Survival could not be estimated in 2015 because of the usual confounding between recapture and survival probabilities in time-structured mark-recapture models. | | | | | | | | |
| --- | --- | --- | --- | --- | --- | --- | --- | --- |
| Sex | Year | Mean | SD | Q_0.025_ | Q_0.25_ | Q_0.5_ | Q_0.75_ | Q_0.975_ |
| Female | 2001 | 0.9570 | 0.0361 | 0.8575 | 0.9442 | 0.9691 | 0.9820 | 0.9900 |
| Female | 2002 | 0.9623 | 0.0292 | 0.8821 | 0.9514 | 0.9719 | 0.9830 | 0.9904 |
| Female | 2003 | 0.9425 | 0.0445 | 0.8279 | 0.9212 | 0.9560 | 0.9765 | 0.9895 |
| Female | 2004 | 0.8456 | 0.0947 | 0.6415 | 0.7811 | 0.8555 | 0.9233 | 0.9847 |
| Female | 2005 | 0.7252 | 0.1158 | 0.5137 | 0.6387 | 0.7195 | 0.8083 | 0.9532 |
| Female | 2006 | 0.8664 | 0.0966 | 0.6363 | 0.8080 | 0.8864 | 0.9454 | 0.9866 |
| Female | 2007 | 0.8294 | 0.1029 | 0.6070 | 0.7601 | 0.8406 | 0.9122 | 0.9829 |
| Female | 2008 | 0.8010 | 0.1011 | 0.6003 | 0.7289 | 0.8043 | 0.8786 | 0.9749 |
| Female | 2009 | 0.9593 | 0.0324 | 0.8711 | 0.9469 | 0.9698 | 0.9820 | 0.9901 |
| Female | 2010 | 0.9632 | 0.0297 | 0.8804 | 0.9534 | 0.9731 | 0.9836 | 0.9904 |
| Female | 2011 | 0.9509 | 0.0499 | 0.8051 | 0.9385 | 0.9701 | 0.9831 | 0.9905 |
| Female | 2012 | 0.5500 | 0.1363 | 0.3326 | 0.4505 | 0.5322 | 0.6324 | 0.8642 |
| Female | 2013 | 0.8986 | 0.1033 | 0.6103 | 0.8603 | 0.9369 | 0.9730 | 0.9892 |
| Female | 2014 | 0.8920 | 0.1284 | 0.5105 | 0.8609 | 0.9445 | 0.9765 | 0.9897 |
| Male | 2001 | 0.9708 | 0.0255 | 0.9012 | 0.9627 | 0.9794 | 0.9881 | 0.9929 |
| Male | 2002 | 0.9746 | 0.0203 | 0.9194 | 0.9674 | 0.9813 | 0.9888 | 0.9930 |
| Male | 2003 | 0.9607 | 0.0315 | 0.8786 | 0.9464 | 0.9705 | 0.9845 | 0.9926 |
| Male | 2004 | 0.8903 | 0.0698 | 0.7364 | 0.8439 | 0.8996 | 0.9474 | 0.9897 |
| Male | 2005 | 0.7947 | 0.0945 | 0.6138 | 0.7270 | 0.7939 | 0.8649 | 0.9697 |
| Male | 2006 | 0.9044 | 0.0736 | 0.7223 | 0.8629 | 0.9219 | 0.9633 | 0.9912 |
| Male | 2007 | 0.8769 | 0.0792 | 0.6981 | 0.8261 | 0.8885 | 0.9403 | 0.9887 |
| Male | 2008 | 0.8554 | 0.0795 | 0.6876 | 0.8010 | 0.8614 | 0.9169 | 0.9830 |
| Male | 2009 | 0.9725 | 0.0224 | 0.9117 | 0.9644 | 0.9799 | 0.9881 | 0.9929 |
| Male | 2010 | 0.9751 | 0.0208 | 0.9173 | 0.9687 | 0.9822 | 0.9892 | 0.9930 |
| Male | 2011 | 0.9667 | 0.0348 | 0.8649 | 0.9591 | 0.9800 | 0.9887 | 0.9930 |
| Male | 2012 | 0.6412 | 0.1247 | 0.4265 | 0.5499 | 0.6317 | 0.7233 | 0.9094 |
| Male | 2013 | 0.9275 | 0.0795 | 0.6975 | 0.9030 | 0.9575 | 0.9821 | 0.9926 |
| Male | 2014 | 0.9209 | 0.1032 | 0.6116 | 0.9037 | 0.9630 | 0.9844 | 0.9928 |

| Table S9. Statistics summarizing samples of 20,000 from the posterior distributions of survival probabilities for polar bears in the Alaska portion of the southern Beaufort Sea in the Ages20+ age class by sex and year, including the mean (Mean), standard deviation (SD), and the 2.5% (Q_0.025_), 25% (Q_0.25_), 50% (Q_0.5_), 75% (Q_0.75_), and 97.5% (Q_0.975_) quantiles. Survival could not be estimated in 2015 because of the usual confounding between recapture and survival probabilities in time-structured mark-recapture models. | | | | | | | | |
| --- | --- | --- | --- | --- | --- | --- | --- | --- |
| Sex | Year | Mean | SD | Q_0.025_ | Q_0.25_ | Q_0.5_ | Q_0.75_ | Q_0.975_ |
| Female | 2001 | 0.8996 | 0.0794 | 0.6859 | 0.8678 | 0.9254 | 0.9561 | 0.9801 |
| Female | 2002 | 0.9105 | 0.0675 | 0.7308 | 0.8819 | 0.9318 | 0.9590 | 0.9808 |
| Female | 2003 | 0.8707 | 0.0927 | 0.6379 | 0.8222 | 0.8961 | 0.9427 | 0.9774 |
| Female | 2004 | 0.7011 | 0.1618 | 0.3840 | 0.5804 | 0.7080 | 0.8323 | 0.9635 |
| Female | 2005 | 0.5314 | 0.1667 | 0.2546 | 0.4083 | 0.5117 | 0.6401 | 0.8970 |
| Female | 2006 | 0.7400 | 0.1579 | 0.4043 | 0.6288 | 0.7577 | 0.8714 | 0.9681 |
| Female | 2007 | 0.6784 | 0.1648 | 0.3597 | 0.5576 | 0.6816 | 0.8089 | 0.9585 |
| Female | 2008 | 0.6317 | 0.1637 | 0.3378 | 0.5079 | 0.6249 | 0.7531 | 0.9441 |
| Female | 2009 | 0.9044 | 0.0721 | 0.7105 | 0.8742 | 0.9270 | 0.9562 | 0.9800 |
| Female | 2010 | 0.9132 | 0.0654 | 0.7353 | 0.8868 | 0.9339 | 0.9598 | 0.9804 |
| Female | 2011 | 0.8894 | 0.1003 | 0.6037 | 0.8549 | 0.9269 | 0.9583 | 0.9811 |
| Female | 2012 | 0.3465 | 0.1470 | 0.1437 | 0.2414 | 0.3177 | 0.4194 | 0.7280 |
| Female | 2013 | 0.7988 | 0.1701 | 0.3687 | 0.7048 | 0.8560 | 0.9348 | 0.9774 |
| Female | 2014 | 0.7955 | 0.1935 | 0.2851 | 0.7057 | 0.8708 | 0.9428 | 0.9786 |
| Male | 2001 | 0.9297 | 0.0595 | 0.7668 | 0.9089 | 0.9491 | 0.9707 | 0.9859 |
| Male | 2002 | 0.9378 | 0.0497 | 0.8023 | 0.9193 | 0.9542 | 0.9726 | 0.9865 |
| Male | 2003 | 0.9083 | 0.0701 | 0.7277 | 0.8747 | 0.9284 | 0.9615 | 0.9844 |
| Male | 2004 | 0.7724 | 0.1338 | 0.4917 | 0.6782 | 0.7855 | 0.8811 | 0.9755 |
| Male | 2005 | 0.6204 | 0.1550 | 0.3378 | 0.5066 | 0.6132 | 0.7302 | 0.9309 |
| Male | 2006 | 0.8031 | 0.1321 | 0.5016 | 0.7176 | 0.8257 | 0.9119 | 0.9786 |
| Male | 2007 | 0.7520 | 0.1408 | 0.4570 | 0.6552 | 0.7628 | 0.8635 | 0.9721 |
| Male | 2008 | 0.7121 | 0.1440 | 0.4259 | 0.6086 | 0.7165 | 0.8237 | 0.9608 |
| Male | 2009 | 0.9335 | 0.0529 | 0.7883 | 0.9138 | 0.9503 | 0.9707 | 0.9859 |
| Male | 2010 | 0.9398 | 0.0481 | 0.8075 | 0.9228 | 0.9556 | 0.9730 | 0.9864 |
| Male | 2011 | 0.9220 | 0.0753 | 0.7019 | 0.9003 | 0.9507 | 0.9719 | 0.9866 |
| Male | 2012 | 0.4347 | 0.1532 | 0.2019 | 0.3226 | 0.4114 | 0.5234 | 0.8091 |
| Male | 2013 | 0.8482 | 0.1420 | 0.4681 | 0.7838 | 0.8997 | 0.9561 | 0.9843 |
| Male | 2014 | 0.8428 | 0.1668 | 0.3751 | 0.7842 | 0.9110 | 0.9613 | 0.9851 |

| Table S10. Statistics summarizing samples of 20,000 from the posterior distributions of survival probabilities for polar bears in the Alaska portion of the southern Beaufort Sea in the Unknown age class by sex and year, including the mean (Mean), standard deviation (SD), and the 2.5% (Q_0.025_), 25% (Q_0.25_), 50% (Q_0.5_), 75% (Q_0.75_), and 97.5% (Q_0.975_) quantiles. Survival could not be estimated in 2015 because of the usual confounding between recapture and survival probabilities in time-structured mark-recapture models. | | | | | | | | |
| --- | --- | --- | --- | --- | --- | --- | --- | --- |
| Sex | Year | Mean | SD | Q_0.025_ | Q_0.25_ | Q_0.5_ | Q_0.75_ | Q_0.975_ |
| Female | 2001 | 0.9205 | 0.0639 | 0.7476 | 0.8961 | 0.9413 | 0.9657 | 0.9833 |
| Female | 2002 | 0.9295 | 0.0535 | 0.7863 | 0.9075 | 0.9465 | 0.9677 | 0.9838 |
| Female | 2003 | 0.8967 | 0.0752 | 0.7050 | 0.8575 | 0.9172 | 0.9548 | 0.9814 |
| Female | 2004 | 0.7526 | 0.1359 | 0.4762 | 0.6549 | 0.7601 | 0.8630 | 0.9698 |
| Female | 2005 | 0.5954 | 0.1536 | 0.3300 | 0.4817 | 0.5814 | 0.6996 | 0.9192 |
| Female | 2006 | 0.7844 | 0.1361 | 0.4894 | 0.6908 | 0.8027 | 0.8978 | 0.9743 |
| Female | 2007 | 0.7304 | 0.1442 | 0.4412 | 0.6253 | 0.7376 | 0.8442 | 0.9676 |
| Female | 2008 | 0.6891 | 0.1438 | 0.4192 | 0.5831 | 0.6867 | 0.7974 | 0.9545 |
| Female | 2009 | 0.9244 | 0.0579 | 0.7678 | 0.9011 | 0.9428 | 0.9653 | 0.9831 |
| Female | 2010 | 0.9312 | 0.0533 | 0.7837 | 0.9116 | 0.9485 | 0.9685 | 0.9838 |
| Female | 2011 | 0.9110 | 0.0840 | 0.6698 | 0.8853 | 0.9429 | 0.9677 | 0.9842 |
| Female | 2012 | 0.4116 | 0.1448 | 0.2020 | 0.3076 | 0.3865 | 0.4881 | 0.7787 |
| Female | 2013 | 0.8347 | 0.1452 | 0.4581 | 0.7594 | 0.8849 | 0.9486 | 0.9810 |
| Female | 2014 | 0.8289 | 0.1723 | 0.3532 | 0.7600 | 0.8979 | 0.9552 | 0.9821 |
| Male | 2001 | 0.9446 | 0.0475 | 0.8157 | 0.9288 | 0.9604 | 0.9770 | 0.9884 |
| Male | 2002 | 0.9513 | 0.0391 | 0.8441 | 0.9371 | 0.9640 | 0.9783 | 0.9886 |
| Male | 2003 | 0.9272 | 0.0564 | 0.7811 | 0.9004 | 0.9438 | 0.9698 | 0.9873 |
| Male | 2004 | 0.8151 | 0.1095 | 0.5791 | 0.7401 | 0.8265 | 0.9033 | 0.9794 |
| Male | 2005 | 0.6787 | 0.1387 | 0.4166 | 0.5783 | 0.6743 | 0.7779 | 0.9477 |
| Male | 2006 | 0.8390 | 0.1114 | 0.5824 | 0.7685 | 0.8603 | 0.9302 | 0.9830 |
| Male | 2007 | 0.7955 | 0.1205 | 0.5373 | 0.7136 | 0.8073 | 0.8910 | 0.9784 |
| Male | 2008 | 0.7612 | 0.1236 | 0.5096 | 0.6741 | 0.7670 | 0.8569 | 0.9698 |
| Male | 2009 | 0.9477 | 0.0422 | 0.8311 | 0.9321 | 0.9615 | 0.9769 | 0.9883 |
| Male | 2010 | 0.9524 | 0.0392 | 0.8436 | 0.9395 | 0.9654 | 0.9790 | 0.9889 |
| Male | 2011 | 0.9377 | 0.0625 | 0.7548 | 0.9213 | 0.9614 | 0.9781 | 0.9890 |
| Male | 2012 | 0.5022 | 0.1461 | 0.2682 | 0.3960 | 0.4836 | 0.5897 | 0.8450 |
| Male | 2013 | 0.8770 | 0.1189 | 0.5518 | 0.8276 | 0.9205 | 0.9655 | 0.9870 |
| Male | 2014 | 0.8698 | 0.1463 | 0.4464 | 0.8264 | 0.9300 | 0.9699 | 0.9877 |

| Table S11. Statistics summarizing samples of 20,000 from the posterior distributions of state transition probabilities of polar bears marked in the Alaska portion of the southern Beaufort Sea, including the mean (Mean), standard deviation (SD), and the 2.5% (Q_0.025_), 25% (Q_0.25_), 50% (Q_0.5_), 75% (Q_0.75_), and 97.5% (Q_0.975_) quantiles. | | | | | | | | |
| --- | --- | --- | --- | --- | --- | --- | --- | --- |
| State from | State to | Mean | SD | Q_0.025_ | Q_0.25_ | Q_0.5_ | Q_0.75_ | Q_0.975_ |
| Nearshore-west | Nearshore-west | 0.3184 | 0.0834 | 0.1717 | 0.2579 | 0.3133 | 0.3733 | 0.4933 |
| Nearshore-west | Nearshore-east | 0.0994 | 0.0331 | 0.0447 | 0.0754 | 0.0960 | 0.1195 | 0.1741 |
| Nearshore-west | Offshore-west | 0.0825 | 0.0526 | 0.0110 | 0.0423 | 0.0727 | 0.1125 | 0.2082 |
| Nearshore-west | Offshore-east | 0.0961 | 0.0358 | 0.0395 | 0.0703 | 0.0918 | 0.1172 | 0.1775 |
| Nearshore-west | Elsewhere | 0.4035 | 0.0883 | 0.2330 | 0.3428 | 0.4033 | 0.4643 | 0.5750 |
| Nearshore-east | Nearshore-west | 0.0262 | 0.0136 | 0.0071 | 0.0162 | 0.0239 | 0.0336 | 0.0594 |
| Nearshore-east | Nearshore-east | 0.5675 | 0.0481 | 0.4676 | 0.5367 | 0.5692 | 0.6009 | 0.6563 |
| Nearshore-east | Offshore-west | 0.0714 | 0.0285 | 0.0265 | 0.0509 | 0.0675 | 0.0882 | 0.1365 |
| Nearshore-east | Offshore-east | 0.1891 | 0.0342 | 0.1267 | 0.1652 | 0.1875 | 0.2113 | 0.2603 |
| Nearshore-east | Elsewhere | 0.1457 | 0.0427 | 0.0731 | 0.1150 | 0.1419 | 0.1726 | 0.2388 |
| Offshore-west | Nearshore-west | 0.4110 | 0.1082 | 0.2131 | 0.3338 | 0.4069 | 0.4841 | 0.6325 |
| Offshore-west | Nearshore-east | 0.0856 | 0.0560 | 0.0111 | 0.0434 | 0.0740 | 0.1163 | 0.2220 |
| Offshore-west | Offshore-west | 0.0685 | 0.0644 | 0.0018 | 0.0208 | 0.0492 | 0.0969 | 0.2380 |
| Offshore-west | Offshore-east | 0.1430 | 0.0658 | 0.0422 | 0.0937 | 0.1344 | 0.1826 | 0.2944 |
| Offshore-west | Elsewhere | 0.2920 | 0.0999 | 0.1175 | 0.2191 | 0.2852 | 0.3576 | 0.5047 |
| Offshore-east | Nearshore-west | 0.0569 | 0.0236 | 0.0204 | 0.0398 | 0.0537 | 0.0707 | 0.1122 |
| Offshore-east | Nearshore-east | 0.2382 | 0.0455 | 0.1553 | 0.2061 | 0.2361 | 0.2680 | 0.3333 |
| Offshore-east | Offshore-west | 0.0428 | 0.0242 | 0.0088 | 0.0251 | 0.0386 | 0.0558 | 0.1011 |
| Offshore-east | Offshore-east | 0.1831 | 0.0446 | 0.1059 | 0.1511 | 0.1802 | 0.2112 | 0.2784 |
| Offshore-east | Elsewhere | 0.4790 | 0.0595 | 0.3633 | 0.4386 | 0.4788 | 0.5194 | 0.5956 |
| Elsewhere | Nearshore-west | 0.0721 | 0.0247 | 0.0304 | 0.0546 | 0.0698 | 0.0871 | 0.1269 |
| Elsewhere | Nearshore-east | 0.0930 | 0.0268 | 0.0452 | 0.0739 | 0.0913 | 0.1104 | 0.1496 |
| Elsewhere | Offshore-west | 0.0966 | 0.0307 | 0.0448 | 0.0747 | 0.0938 | 0.1152 | 0.1648 |
| Elsewhere | Offshore-east | 0.1147 | 0.0299 | 0.0618 | 0.0936 | 0.1127 | 0.1336 | 0.1791 |
| Elsewhere | Elsewhere | 0.6235 | 0.0463 | 0.5291 | 0.5928 | 0.6245 | 0.6553 | 0.7111 |

| Table S12. Statistics summarizing samples of 20,000 from the posterior distributions of the proportion of polar bears marked in the Alaska portion of the southern Beaufort Sea estimated to be alive that are in one of the four spatial states in which sampling effort occurs (states 1-4), by year, including the mean (Mean), standard deviation (SD), and the 2.5% (Q_0.025_), 25% (Q_0.25_), 50% (Q_0.5_), 75% (Q_0.75_), and 97.5% (Q_0.975_) quantiles. The proportion could not be estimated in 2016 because of the usual confounding between recapture and survival probabilities in time-structured mark-recapture models. | | | | | | | |
| --- | --- | --- | --- | --- | --- | --- | --- |
| Year | Mean | SD | Q_0.025_ | Q_0.25_ | Q_0.5_ | Q_0.75_ | Q_0.975_ |
| 2002 | 0.8850 | 0.0297 | 0.8231 | 0.8655 | 0.8862 | 0.9063 | 0.9385 |
| 2003 | 0.7940 | 0.0321 | 0.7292 | 0.7723 | 0.7941 | 0.8159 | 0.8557 |
| 2004 | 0.7481 | 0.0319 | 0.6846 | 0.7267 | 0.7483 | 0.7698 | 0.8097 |
| 2005 | 0.6818 | 0.0393 | 0.6052 | 0.6549 | 0.6818 | 0.7085 | 0.7582 |
| 2006 | 0.6845 | 0.0401 | 0.6047 | 0.6576 | 0.6846 | 0.7116 | 0.7635 |
| 2007 | 0.6783 | 0.0403 | 0.5992 | 0.6510 | 0.6783 | 0.7056 | 0.7566 |
| 2008 | 0.6883 | 0.0401 | 0.6095 | 0.6617 | 0.6882 | 0.7154 | 0.7671 |
| 2009 | 0.6976 | 0.0400 | 0.6175 | 0.6706 | 0.6983 | 0.7253 | 0.7746 |
| 2010 | 0.6718 | 0.0412 | 0.5890 | 0.6445 | 0.6720 | 0.6997 | 0.7510 |
| 2011 | 0.6729 | 0.0389 | 0.5950 | 0.6469 | 0.6735 | 0.6993 | 0.7483 |
| 2012 | 0.6698 | 0.0395 | 0.5917 | 0.6433 | 0.6701 | 0.6967 | 0.7458 |
| 2013 | 0.6926 | 0.0442 | 0.6058 | 0.6627 | 0.6926 | 0.7228 | 0.7789 |
| 2014 | 0.6347 | 0.0462 | 0.5448 | 0.6030 | 0.6344 | 0.6655 | 0.7265 |
| 2015 | 0.6225 | 0.0468 | 0.5326 | 0.5905 | 0.6223 | 0.6538 | 0.7163 |

| Table S13. Statistics summarizing samples of 20,000 from the posterior distributions of recapture probabilities for polar bears in the Alaska portion of the southern Beaufort Sea, by spatial state and year, including the mean (Mean), standard deviation (SD), and the 2.5% (Q_0.025_), 25% (Q_0.25_), 50% (Q_0.5_), 75% (Q_0.75_), and 97.5% (Q_0.975_) quantiles. Recapture probability could not be estimated in 2016 because of the usual confounding between recapture and survival probabilities in time-structured mark-recapture models. | | | | | | | | |
| --- | --- | --- | --- | --- | --- | --- | --- | --- |
| State | Year | Mean | SD | Q_0.025_ | Q_0.25_ | Q_0.5_ | Q_0.75_ | Q_0.975_ |
| Nearshore-west | 2002 | 0.2452 | 0.1012 | 0.0901 | 0.1703 | 0.2306 | 0.3061 | 0.4806 |
| Nearshore-west | 2003 | 0.1573 | 0.0679 | 0.0592 | 0.1087 | 0.1455 | 0.1930 | 0.3246 |
| Nearshore-west | 2004 | 0.2581 | 0.0817 | 0.1301 | 0.2000 | 0.2469 | 0.3054 | 0.4466 |
| Nearshore-west | 2005 | 0.2193 | 0.0776 | 0.1034 | 0.1633 | 0.2072 | 0.2613 | 0.4030 |
| Nearshore-west | 2006 | 0.3516 | 0.0955 | 0.1913 | 0.2825 | 0.3427 | 0.4107 | 0.5611 |
| Nearshore-west | 2007 | 0.2422 | 0.0819 | 0.1170 | 0.1835 | 0.2301 | 0.2887 | 0.4362 |
| Nearshore-west | 2008 | 0.2627 | 0.0839 | 0.1321 | 0.2018 | 0.2504 | 0.3126 | 0.4532 |
| Nearshore-west | 2009 | 0.3797 | 0.1044 | 0.2056 | 0.3043 | 0.3688 | 0.4439 | 0.6125 |
| Nearshore-west | 2010 | 0.2570 | 0.0815 | 0.1304 | 0.1983 | 0.2451 | 0.3037 | 0.4482 |
| Nearshore-west | 2011 | 0.3168 | 0.0896 | 0.1727 | 0.2523 | 0.3053 | 0.3702 | 0.5229 |
| Nearshore-west | 2012 | 0.3038 | 0.0839 | 0.1678 | 0.2433 | 0.2945 | 0.3537 | 0.4922 |
| Nearshore-west | 2013 | 0.3848 | 0.1216 | 0.1831 | 0.2947 | 0.3722 | 0.4628 | 0.6513 |
| Nearshore-west | 2014 | 0.2259 | 0.0941 | 0.0940 | 0.1582 | 0.2085 | 0.2736 | 0.4587 |
| Nearshore-west | 2015 | 0.1462 | 0.0766 | 0.0504 | 0.0936 | 0.1296 | 0.1787 | 0.3417 |
| Nearshore-east | 2002 | 0.2175 | 0.0746 | 0.0955 | 0.1635 | 0.2100 | 0.2632 | 0.3841 |
| Nearshore-east | 2003 | 0.1369 | 0.0467 | 0.0611 | 0.1033 | 0.1316 | 0.1644 | 0.2433 |
| Nearshore-east | 2004 | 0.2302 | 0.0545 | 0.1375 | 0.1917 | 0.2254 | 0.2628 | 0.3501 |
| Nearshore-east | 2005 | 0.1936 | 0.0512 | 0.1099 | 0.1568 | 0.1878 | 0.2247 | 0.3090 |
| Nearshore-east | 2006 | 0.3204 | 0.0726 | 0.1947 | 0.2688 | 0.3143 | 0.3665 | 0.4763 |
| Nearshore-east | 2007 | 0.2149 | 0.0537 | 0.1248 | 0.1768 | 0.2095 | 0.2470 | 0.3333 |
| Nearshore-east | 2008 | 0.2344 | 0.0559 | 0.1393 | 0.1945 | 0.2296 | 0.2692 | 0.3569 |
| Nearshore-east | 2009 | 0.3464 | 0.0649 | 0.2318 | 0.3008 | 0.3417 | 0.3877 | 0.4849 |
| Nearshore-east | 2010 | 0.2281 | 0.0471 | 0.1457 | 0.1948 | 0.2246 | 0.2573 | 0.3312 |
| Nearshore-east | 2011 | 0.2852 | 0.0524 | 0.1941 | 0.2478 | 0.2813 | 0.3184 | 0.3984 |
| Nearshore-east | 2012 | 0.2734 | 0.0539 | 0.1819 | 0.2357 | 0.2688 | 0.3063 | 0.3922 |
| Nearshore-east | 2013 | 0.3527 | 0.0955 | 0.1902 | 0.2834 | 0.3439 | 0.4138 | 0.5613 |
| Nearshore-east | 2014 | 0.1988 | 0.0626 | 0.1012 | 0.1541 | 0.1905 | 0.2336 | 0.3424 |
| Nearshore-east | 2015 | 0.1263 | 0.0551 | 0.0540 | 0.0895 | 0.1162 | 0.1506 | 0.2597 |
| Offshore-west | 2002 | 0.0487 | 0.0292 | 0.0127 | 0.0281 | 0.0419 | 0.0621 | 0.1240 |
| Offshore-west | 2003 | 0.0280 | 0.0162 | 0.0077 | 0.0167 | 0.0244 | 0.0354 | 0.0697 |
| Offshore-west | 2004 | 0.0511 | 0.0252 | 0.0176 | 0.0334 | 0.0460 | 0.0634 | 0.1142 |
| Offshore-west | 2005 | 0.0416 | 0.0216 | 0.0137 | 0.0265 | 0.0370 | 0.0515 | 0.0956 |
| Offshore-west | 2006 | 0.0784 | 0.0377 | 0.0273 | 0.0517 | 0.0710 | 0.0970 | 0.1720 |
| Offshore-west | 2007 | 0.0470 | 0.0235 | 0.0162 | 0.0304 | 0.0421 | 0.0582 | 0.1046 |
| Offshore-west | 2008 | 0.0524 | 0.0260 | 0.0178 | 0.0339 | 0.0471 | 0.0647 | 0.1169 |
| Offshore-west | 2009 | 0.0872 | 0.0401 | 0.0320 | 0.0587 | 0.0796 | 0.1077 | 0.1861 |
| Offshore-west | 2010 | 0.0505 | 0.0240 | 0.0180 | 0.0335 | 0.0459 | 0.0622 | 0.1102 |
| Offshore-west | 2011 | 0.0669 | 0.0307 | 0.0247 | 0.0453 | 0.0611 | 0.0819 | 0.1428 |
| Offshore-west | 2012 | 0.0630 | 0.0284 | 0.0242 | 0.0429 | 0.0578 | 0.0775 | 0.1318 |
| Offshore-west | 2013 | 0.0914 | 0.0501 | 0.0278 | 0.0559 | 0.0804 | 0.1142 | 0.2194 |
| Offshore-west | 2014 | 0.0434 | 0.0254 | 0.0129 | 0.0261 | 0.0376 | 0.0541 | 0.1078 |
| Offshore-west | 2015 | 0.0260 | 0.0184 | 0.0067 | 0.0143 | 0.0215 | 0.0320 | 0.0719 |
| Offshore-east | 2002 | 0.1933 | 0.0690 | 0.0819 | 0.1426 | 0.1856 | 0.2343 | 0.3493 |
| Offshore-east | 2003 | 0.1199 | 0.0418 | 0.0530 | 0.0898 | 0.1149 | 0.1445 | 0.2158 |
| Offshore-east | 2004 | 0.2044 | 0.0502 | 0.1197 | 0.1691 | 0.1993 | 0.2348 | 0.3149 |
| Offshore-east | 2005 | 0.1710 | 0.0466 | 0.0958 | 0.1371 | 0.1659 | 0.1990 | 0.2760 |
| Offshore-east | 2006 | 0.2884 | 0.0683 | 0.1714 | 0.2399 | 0.2822 | 0.3312 | 0.4378 |
| Offshore-east | 2007 | 0.1905 | 0.0496 | 0.1079 | 0.1552 | 0.1856 | 0.2204 | 0.3015 |
| Offshore-east | 2008 | 0.2084 | 0.0524 | 0.1198 | 0.1712 | 0.2037 | 0.2407 | 0.3233 |
| Offshore-east | 2009 | 0.3132 | 0.0636 | 0.2027 | 0.2686 | 0.3087 | 0.3531 | 0.4505 |
| Offshore-east | 2010 | 0.2026 | 0.0445 | 0.1249 | 0.1713 | 0.1990 | 0.2303 | 0.3012 |
| Offshore-east | 2011 | 0.2555 | 0.0511 | 0.1655 | 0.2191 | 0.2522 | 0.2881 | 0.3654 |
| Offshore-east | 2012 | 0.2444 | 0.0514 | 0.1568 | 0.2083 | 0.2399 | 0.2749 | 0.3587 |
| Offshore-east | 2013 | 0.3194 | 0.0903 | 0.1697 | 0.2538 | 0.3101 | 0.3748 | 0.5202 |
| Offshore-east | 2014 | 0.1758 | 0.0569 | 0.0880 | 0.1355 | 0.1678 | 0.2070 | 0.3069 |
| Offshore-east | 2015 | 0.1107 | 0.0501 | 0.0467 | 0.0775 | 0.1011 | 0.1321 | 0.2324 |

| Table S14. Statistics summarizing samples of 20,000 from the posterior distributions of annual abundance estimates for polar bears of the Alaska portion of the southern Beaufort Sea, including the mean (Mean), standard deviation (SD), and the 2.5% (Q_0.025_), 25% (Q_0.25_), 50% (Q_0.5_), 75% (Q_0.75_), and 97.5% (Q_0.975_) quantiles. Abundance could not be estimated in 2016 because of the usual confounding between recapture and survival probabilities in time-structured mark-recapture models. | | | | | | | |
| --- | --- | --- | --- | --- | --- | --- | --- |
|  |  |  |  |  |  |  |  |
| Year | Mean | SD | Q_0.025_ | Q_0.25_ | Q_0.5_ | Q_0.75_ | Q_0.975_ |
| 2002 | 663 | 302 | 305 | 463 | 594 | 786 | 1422 |
| 2003 | 1310 | 522 | 644 | 950 | 1197 | 1538 | 2628 |
| 2004 | 963 | 240 | 600 | 794 | 929 | 1093 | 1535 |
| 2005 | 924 | 280 | 514 | 727 | 879 | 1069 | 1591 |
| 2006 | 524 | 131 | 328 | 430 | 503 | 594 | 835 |
| 2007 | 655 | 167 | 399 | 538 | 632 | 748 | 1045 |
| 2008 | 560 | 136 | 352 | 464 | 542 | 636 | 876 |
| 2009 | 413 | 76 | 290 | 359 | 404 | 458 | 587 |
| 2010 | 562 | 130 | 363 | 471 | 543 | 633 | 873 |
| 2011 | 597 | 110 | 419 | 519 | 585 | 662 | 845 |
| 2012 | 754 | 168 | 495 | 636 | 731 | 845 | 1154 |
| 2013 | 487 | 165 | 257 | 370 | 457 | 571 | 897 |
| 2014 | 521 | 176 | 262 | 396 | 493 | 611 | 943 |
| 2015 | 573 | 233 | 232 | 410 | 533 | 689 | 1140 |


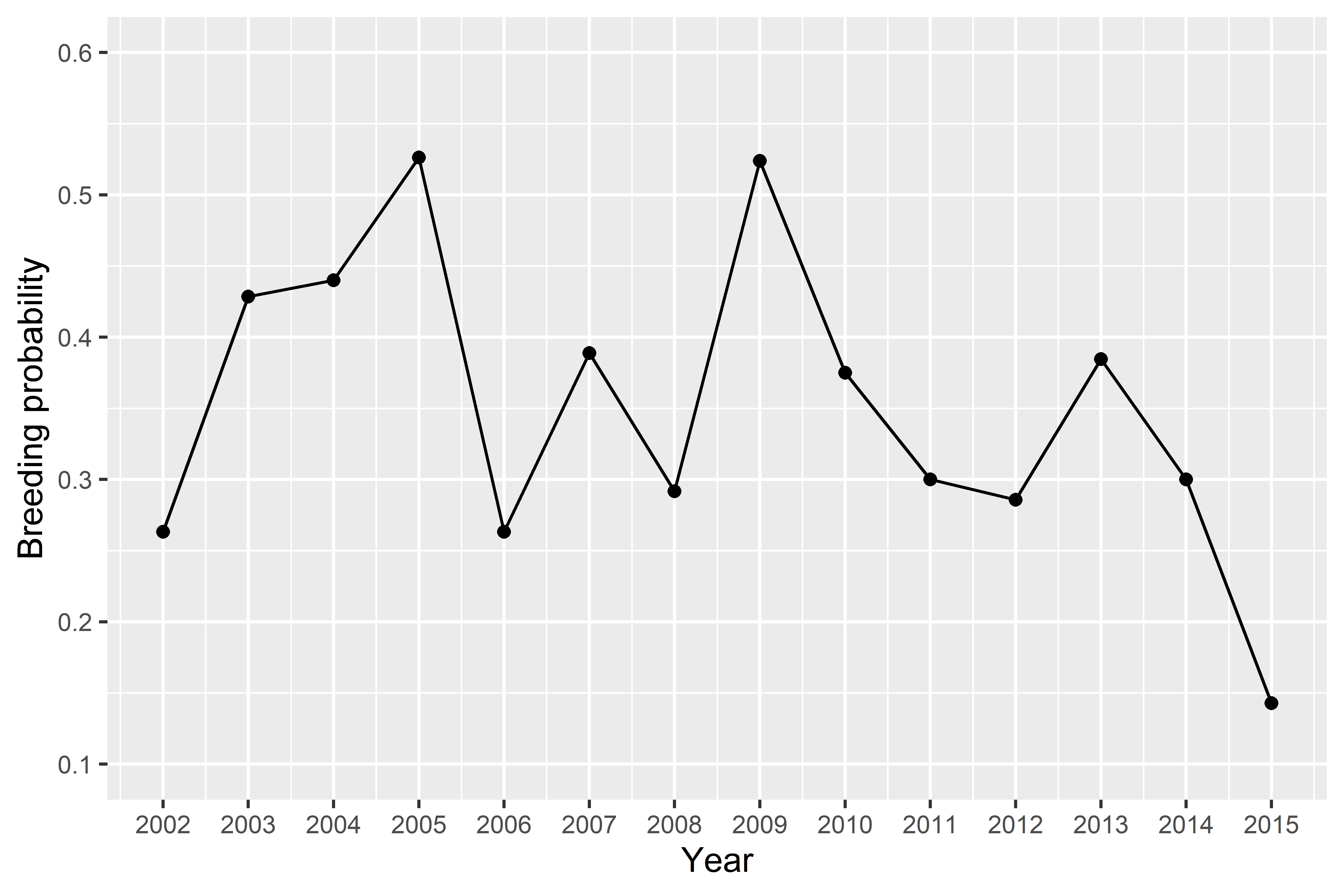


Figure S1. Annual breeding probabilities used to establish new litters of age-0 cubs in the population projections.


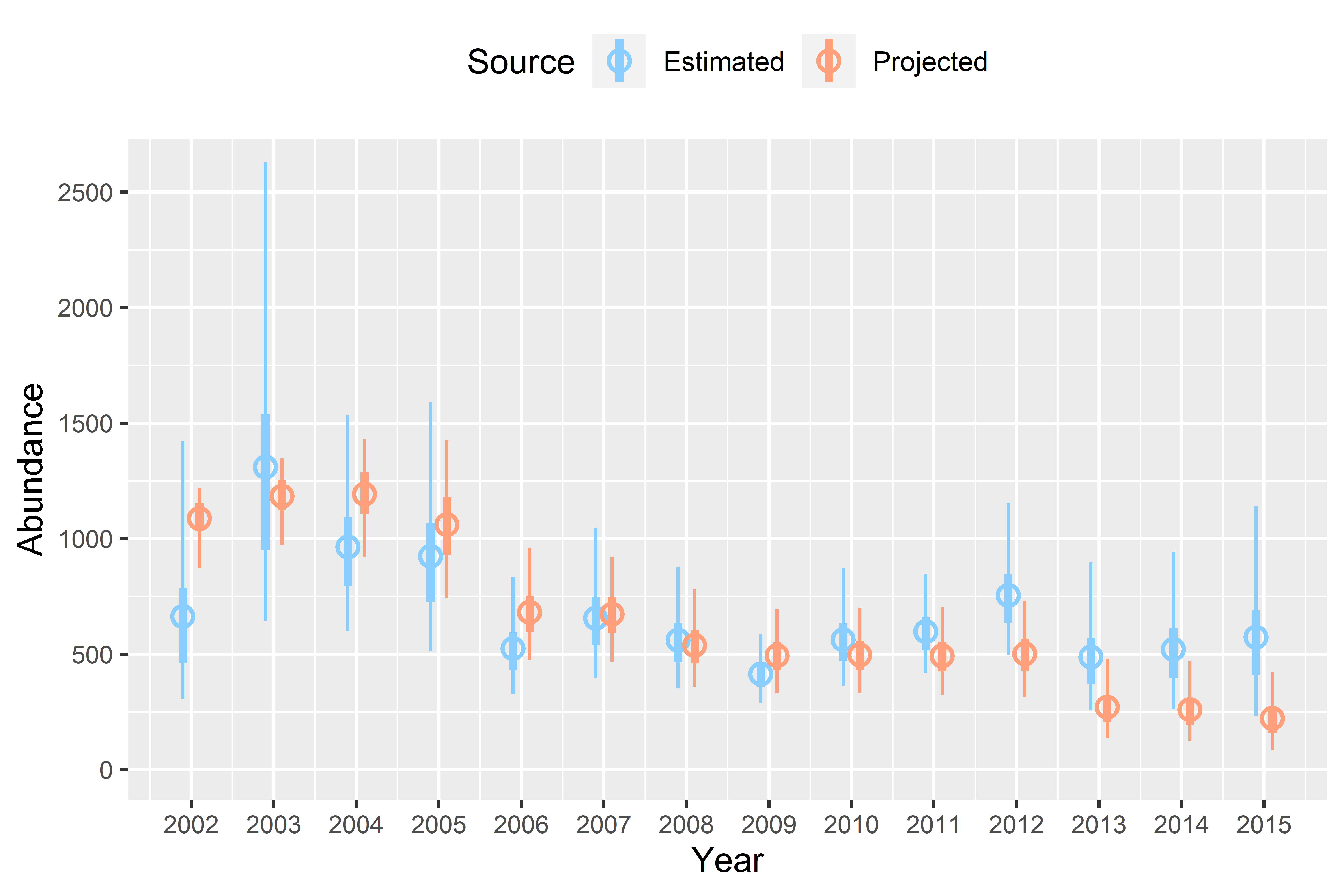


Figure S2. Annual abundance estimates and scaled abundance projections. The open circles represent means and the thick and thin lines represent 50% and 95% credible intervals for the abundance estimates and the central 50% and 95% of the 20,000 scaled abundance projections, respectively.
